# Validation of the DIGEST-FEES as a Global Outcome Measure for Pharyngeal Dysphagia in Parkinson’s Disease

**DOI:** 10.1007/s00455-023-10650-6

**Published:** 2023-12-22

**Authors:** Bendix Labeit, Sriramya Lapa, Paul Muhle, Sonja Suntrup-Krueger, Inga Claus, Florin Gandor, Sigrid Ahring, Stephan Oelenberg, Rainer Dziewas, Tobias Warnecke

**Affiliations:** 1 Department of Neurology, Medical Faculty and University Hospital Düsseldorf, Heinrich Heine University Düsseldorf, Moorenstraße 5, 40225 Düsseldorf, Germany; 2 Department of Neurology, University Hospital Frankfurt, Goethe University, Frankfurt am Main, Germany; 3 Department of Neurology with Institute of Translational Neurology, University Hospital Muenster, Muenster, Germany; 4 Movement Disorders Clinic, Beelitz-Heilstätten, Germany; 5 Department of Neurology, Otto-von-Guericke University, Magdeburg, Germany; 6 Department of Neurology and Neurorehabilitation, Klinikum Osnabrueck – Academic Teaching Hospital of the WWU Muenster, Osnabrueck, Germany

**Keywords:** DIGEST, Oropharyngeal dysphagia, Parkinson, Aspiration, Pneumonia, Malnutrition

## Abstract

Flexible Endoscopic Evaluation of Swallowing (FEES) is one of two diagnostic gold standards for pharyngeal dysphagia in Parkinson's disease (PD), however, validated global outcome measures at the patient level are widely lacking. The Dynamic Imaging Grade of Swallowing Toxicity for Flexible Endoscopic Evaluation of Swallowing (DIGEST-FEES) represents such an outcome measure but has been validated primarily for head and neck cancer collectives. The objective of this study was, therefore, to investigate the validity of the DIGEST-FEES in patients with PD. Content validity was evaluated with a modified Delphi expert survey. Subsequently, 66 FEES videos in PD patients were scored with the DIGEST-FEES. Criterion validity was determined using Spearman's correlation coefficient between the DIGEST-FEES and the Penetration-Aspiration Scale (PAS), the Yale-Residue-Rating-Scale, the Functional-Oral-Intake-Scale (FOIS), and the swallowing-related Unified-Parkinson-Disease-Rating-Scale (UPDRS) items. Inter-rater reliability was determined using 10 randomly selected FEES-videos examined by a second rater. As a result, the overall DIGEST-FEES-rating exhibited significant correlations with the Yale-Valleculae-Residue-Scale (*r* = 0.84; *p* < 0.001), the Yale-Pyriform-Sinus-Residue-Scale (*r* = 0.70; *p* < 0.001), the FOIS (*r* =  − 0.55, *p* < 0.001), and the UPDRS-Swallowing-Item-Score (*r* = 0.42, *p* < 0.001). Further, the DIGEST-FEES-safety subscore correlated with the PAS (*r* = 0.63, *p* < 0.001). Inter-rater reliability was high for the overall DIGEST-FEES rating (quadratic weighted kappa of 0.82). Therefore, DIGEST-FEES is a valid and reliable score to evaluate overall pharyngeal dysphagia severity in PD. Nevertheless, the modified Delphi survey identified domains where DIGEST-FEES may need to be specifically adapted to PD or neurological collectives in the future.

**Supplementary Information:**

The online version contains supplementary material available at 10.1007/s00455-023-10650-6.

## Introduction

Oropharyngeal dysphagia is a common and complication-prone condition in Parkinson’s disease (PD), affecting approximately 82% of patients when instrumental diagnostics are used [1]. Although longer duration and more severe stage of the disease are associated with dysphagia [2], swallowing difficulties or alterations may also occur in mild disease stages or as a prodromal condition [1, 3–5]. Dysphagia may lead to serious complications such as malnutrition and aspiration pneumonia [6], and thus can result in adverse functional outcomes, hospitalization and increased mortality [7, 8]. In addition, dysphagia can lead to difficulty in medication adherence, and therefore, may also be involved in the development of motor fluctuations [9]. However, dysphagia not only causes medical complications, but often affects patient quality of life [10, 11], which can be assessed using validated patient-reported outcome measures. The diagnostic work-up is complicated by the fact that silent penetration and aspiration may occur that are neither noticed by the patient nor detected during a clinical swallowing examination [12]. Therefore, instrumental dysphagia diagnostics that visualize swallowing are needed to increase diagnostic sensitivity [1] and characterize the dysphagia phenotype [13], e.g. the dysphagia mechanism. Both, Videofluoroscopic Swallowing Study (VFSS) and Flexible Endoscopic Evaluation of Swallowing (FEES) are considered diagnostic gold standards and can be used equally to detect swallowing pathophysiology resulting in penetration, aspiration, and/or pharyngeal residue [14]. In patients with PD, FEES without radiation exposure has the distinct advantage of allowing longer examination protocols and repeated examinations that may be required due to fluctuating medication effects. However, despite the high clinical relevance, there is a lack of valid visuoperceptual scores with good psychometric parameters to classify the severity of pharyngeal dysphagia in instrumental assessment [15].

A promising tool in this area is the Dynamic Imaging Grade of Swallowing Toxicity (DIGEST), which was originally developed for head and neck cancer patients [16]. This five-point ordinal score assesses the global severity of pharyngeal dysphagia at the patient level (rather than at the level of individual swallows) and considers the two dimensions of swallowing safety and swallowing efficiency. Swallowing safety is graded using the Penetration-Aspiration Scale (PAS), considering the frequency and quantity of the events. Swallowing efficiency is graded based on the amount of pharyngeal residue, taking into account the bolus consistency with which they occurred. This tool has since been adapted and validated for FEES [17]. Although the DIGEST has been used in neurological patients in individual studies (e.g. PD [18], amyotrophic lateral sclerosis [19, 20], and oculopharyngeal muscular dystrophy [21]), no comprehensive validation study has been conducted in this patient population, and to our knowledge, no endoscopic study has used the DIGEST-FEES. Specifically, PD patients may have unique dysphagia mechanisms such as oropharyngeal freezing [22], oropharyngeal bradykinesia [23], alterations in respiratory-swallow coordination [24, 25], lingual pressure dysfunction [26–28] or decreased capacity for cognitive cortical processing [29]. Therefore, the underlying pathology of dysphagia is considerably different from that seen in patients with head and neck cancer, which may also have implications for severity classification. The aim of this study was therefore to investigate whether DIGEST-FEES is suitable for pharyngeal dysphagia in PD patients and to identify potential problem areas that may need to be considered in the future adaptation of the tool in this patient group. For this purpose, a modified Delphi survey of experts in the field of dysphagia in PD was conducted. Subsequently, 66 FEES videos of PD patients were scored with the DIGEST-FEES and correlated with frequently used outcome parameters.

## Methods

### Modified Delphi Expert Survey

An expert panel of 10 clinician scientists (the authors of this paper) participated in the survey. Each panel member holds the FEES instructor certificate issued by the German Society for Dysphagia/the European Society for Swallowing Disorders [30]. Seven of the participants are neurology physicians, three of the participants are speech-language pathologists. All participating experts have a strong publication record in peer-reviewed journals focusing on dysphagia in PD.

A total of 15 questions were posed to the participants, addressing the utilization of DIGEST-FEES in patients with Parkinson's disease (PD) (and identical questions about its use in neurogenic dysphagia in general), with a specific focus on content validity. The participants were asked to rate their agreement or disagreement on a five-point scale for each question, ranging from ‘strongly agree’ to ‘strongly disagree.’ Additionally, participants were given the opportunity to provide comments, including reasons for their responses, in a text box following each question. The questionnaire used in the study is provided as Supplementary Material 1. In the second round of the Delphi process, the results of the initial round, including participant comments, were presented. The responses to the five-point scale questions were depicted in pie charts, while the comments were presented as free text. Participants were once again asked the same set of questions as in the first round. The survey was concluded upon achieving significant convergence of responses, indicated by more than 60% of participants consistently selecting adjacent response scale levels, such as 'strongly agree' and 'agree'.

### Swallowing Assessment

FEES videos from 66 PD patients collected as part of another study [14] were used for the swallowing assessment. All patients were diagnosed with idiopathic PD according to the British Parkinson's Society Brain Bank criteria or the revised Movement Disorder Society (MDS) criteria [31, 32]. Furthermore, inclusion criteria necessitated a Hoehn and Yahr disease stage of ≥ 2. Exclusion criteria encompassed the need for a feeding tube, the presence of other dysphagia-related conditions (e.g. stroke, neuromuscular diseases, neurodegenerative diseases other than PD, head and neck cancer, or structural abnormalities in the oropharyngolaryngeal region), and the utilization of deep brain stimulation and/or intestinal or subcutaneous continuous pump therapy. FEES was performed according to a standardized protocol with testing of three different food consistencies in the following order: 8 ml of green jelly (semi-solid), 5 ml blue-dyed liquid, and white bread (solid) with a size of approximately 3 × 3 × 0.5 cm. Each consistency was tested in 3 swallowing trials. In addition to the DIGEST-FEES, the maximum PAS [33], the maximum Yale-Valleculae-Residue-Scale [34], and the maximum Yale-Pyriform-Sinus-Residue-Scale [34] during the examination were scored and the Functional Oral Intake Scale (FOIS) [35] was recorded. As subjective parameter of impairment, the sum of the 2 swallowing-related questions (questions 2.3 and 2.4) of the United-Parkinson Disease Rating Scale (UPDRS) were noted. To determine inter-rater reliability, the DIGEST-FEES was assessed in 10 randomly selected subjects by a second rater. In addition, intra-rater reliability was determined in 10 randomly selected subjects by redetermining DIGEST-FEES by the same rater 3 weeks after the first assessment.

### Statistical Analysis

Spearman’s correlation coefficient was used to assess the relation between the DIGEST-FEES (as well as the subdomains for swallowing safety and swallowing efficiency) and the maximum PAS, the maximum Yale-Residue-Scale, the FOIS, and the sum score of the swallowing-related items of the UPDRS. Due to conducting multiple tests totaling 15, the significance threshold was adjusted to *p* < 0.05/15, resulting in a criterion of *p* < 0.003 to establish statistical significance. Inter-rater reliability as well as intra-rater reliability was determined using the quadratic weighted kappa coefficient.

## Results

### Content Validity in the Delphi Survey

Two Delphi rounds were completed by a total of 10 experts. After that, no further round was conducted, as there was a high degree of agreement between the experts.

All experts agreed or strongly agreed, that impairment of swallowing safety and impairment in efficiency of bolus clearance are parameters that comprehensively characterize clinically relevant pharyngeal dysphagia in patients with PD, whereas 90% agreed that this also applies to neurogenic dysphagia in general. Further, all experts agreed or strongly agreed that the PAS is suitable as basis for the classification of swallowing safety in PD and neurogenic dysphagia. The subdivision of PAS scores into 1/2 (normal); 3/4; 5/6 and 7/8 was considered suitable for PD patients and for neurogenic dysphagia by all experts (agreement or strong agreement). Similarly, all experts agreed or strongly agreed on the usefulness of the dimension of frequency in PAS events (“single event”, “intermittent”, and “chronic”) for both PD and neurogenic dysphagia. 90% of experts agreed or strongly agreed with the usefulness of the distinction between “gross” and “non-gross” penetration and aspiration in PD, whereas all experts agreed in neurogenic dysphagia. Overall categorization of impaired swallowing safety was considered useful by each 90% of experts in both PD patients and neurogenic dysphagia (agreement or strong agreement).

The assessment of swallowing efficiency based on the maximum percentage of pharyngeal bolus residue was considered useful by 90% of experts in PD and by all experts in neurogenic dysphagia (agreement or strong agreement). Similarly, 90% of experts agreed or strongly agreed on the classification that residue of less than 10% are considered clinically not relevant in both PD and neurogenic dysphagia. The classification of 10–33% residue as mild, 33–66% as moderate, and over 66% as severe was considered useful by all experts for both PD and neurogenic dysphagia (agreement or strong agreement). Consideration of consistencies in the classification of swallowing efficiency, with a higher degree of impairment when residue are not present only at solid consistencies, was agreed with or strongly agreed with by 80% of the experts in PD and 90% in neurogenic dysphagia. However, there were also dissenting votes on this topic. The same applied to the rating of a higher degree of impairment when residue occur at all consistencies. Here each 80% of the experts agreed or strongly agreed in both PD and in neurogenic dysphagia. Accordingly, the majority of 90% of experts agreed or strongly agreed that the overall classification of impaired swallowing efficiency is appropriate for both PD patients and for neurogenic dysphagia.

The intended interaction between impaired swallowing efficiency and impaired swallowing safety in determining overall pharyngeal dysphagia severity was considered useful by 80% of experts for PD and 90% of experts for neurogenic dysphagia (agreement or strong agreement), with individual dissenting voices. The results of the first and second Delphi-round in detail are provided in Supplementary Material 2 and 3.

### Description of the Patient Cohort

According to the exclusion criteria (no tube feeding), our cohort included mostly patients with mild impairment of FOIS (7 to 4, with no impairment of oral intake in ~ 63% of patients). Only a few patients (~ 12%) had penetration and aspiration, whereas the majority of patients (> 60%) had impaired swallowing efficiency. A detailed description of the cohort can be found in Table 1.

**Table 1:** Description of the patient cohort

Mean age in years ± SD	68.4 ± 8.8
Sex m/f	44/22
Hoehn and Yahr Stage 2, *n* (%)	29 (43.9%)
Hoehn and Yahr Stage 2.5, *n* (%)	10 (15.2%)
Hoehn and Yahr Stage 3, *n* (%)	17 (25.8%)
Hoehn and Yahr Stage 4, *n* (%)	9 (13.6%)
Hoehn and Yahr Stage 5, *n* (%)	1 (1.5%)
DIGEST-safety 0, *n* (%)	63 (95.5%)
DIGEST-safety 1, *n* (%)	1 (1.5%)
DIGEST-safety 2, *n* (%)	1 (1.5%)
DIGEST-safety 3, *n* (%)	1 (1.5%)
DIGEST-efficiency 0, *n* (%)	24 (37.9%)
DIGEST-efficiency 1, *n* (%)	7 (10.6%)
DIGEST-efficiency 2, *n* (%)	14 (21.2%)
DIGEST-efficiency 3, *n* (%)	17 (25.8%)
DIGEST-efficiency 4, *n* (%)	3 (4.5%)
DIGEST-FEES 0, *n* (%)	25 (37.9%)
DIGEST-FEES 1, *n* (%)	21 (31.8%)
DIGEST-FEES 2, *n* (%)	17 (25.8%)
DIGEST-FEES 3, *n* (%)	3 (4.5%)
PAS 1, *n* (%)	58 (87.9%)
PAS 2, *n* (%)	2 (3.0%)
PAS 3, *n* (%)	4 (6.1%)
PAS 6, *n* (%)	1 (1.5%)
PAS 7, *n* (%)	1 (1.5%)
Yale residue valleculae 1, *n* (%)	6 (9.1%)
Yale residue valleculae 2, *n* (%)	18 (27.3%)
Yale residue valleculae 3, *n* (%)	10 (15.2%)
Yale residue valleculae 4, *n* (%)	20 (30.3%)
Yale residue valleculae 5, *n* (%)	12 (18.2%)
Yale residue pyriform sinus 1, *n* (%)	29 (43.9%)
Yale residue pyriform sinus 2, *n* (%)	22 (33.3%)
Yale residue pyriform sinus 3, *n* (%)	7 (10.6%)
Yale residue pyriform sinus 4, *n* (%)	5 (7.6%)
Yale residue pyriform sinus 5, *n* (%)	3 (4.5%)
FOIS 7, *n* (%)	42 (63.6%)
FOIS 6, *n* (%)	12 (18.2%)
FOIS 5, *n* (%)	9 (13.6%)
FOIS 4, *n* (%)	3 (4.5%)
UPDRS swallow 0, *n* (%)	36 (54.5%)
UPDRS swallow 1, *n* (%)	12 (18.2%)
UPDRS swallow 2, *n* (%)	9 (13.6%)
UPDRS swallow 3, *n* (%)	3 (4.5%)
UPDRS swallow 4, *n* (%)	2 (3.0%)
UPDRS swallow 5, *n* (%)	2 (3.0%)
UPDRS swallow 6, *n* (%)	1 (1.5%)

*SD* Standard deviation,* m* male,* f* female, *DIGEST* Dynamic Imaging Grade of Swallowing Toxicity, *DIGEST-FEES* Dynamic Imaging Grade of Swallowing Toxicity for Flexible Endoscopic Evaluation of Swallowing, *PAS* Penetration-Aspiration-Scale, *FOIS* Functional-Oral-Intake-Scale, *UPDRS* swallow sum score of the swallowing related United-Parkinson-Disease-Rating-Scale items

### Criterion Validity

As a measure of criterion validity, the DIGEST-FEES and the subscores for swallowing safety and swallowing efficiency were correlated with the PAS, the Yale-Residue-Scale, the sum score of the UPDRS swallowing-related questions, and the FOIS. There was a significant correlation between DIGEST-FEES and between the subscore of swallowing efficiency and the Yale-Residue-Valleculae-Scale and the Yale-Residue-Pyriform-Sinus-Scale. Further, there was a significant correlation between the DIGEST-FEES and both of the subscores and the score on the UPDRS items on swallowing. In addition, there was an inverse correlation between the DIGEST-FEES (and its subscores) and the FOIS. While there was no correlation between the PAS and the DIGEST-FEES, PAS was related to the safety subscore of DIGEST-FEES. The results of the correlation analysis with the correlation coefficient and the *p* value are shown in Table 2.

**Table 2:** Spearman’s correlation coefficient for the association of different criterion validity measures and the Dynamic Imaging Grade of Swallowing Toxicity for Flexible Endoscopic Evaluation of Swallowing (DIGEST-FEES)

Measure	Safety	Efficiency	DIGEST-FEES
PAS	0.63	0.21	0.20
	***p* < 0.001**	*p* = 0.086	*p* = 0.110
Yale residue vallecuale	0.32	0.87	0.84
	*p* = 0.009	***p* < 0.001**	***p* < 0.001**
Yale residue pyrifom sinus	0.29	0.74	0.70
	*p* = 0.019	***p* < 0.001**	***p* < 0.001**
FOIS	− 0.40	− 0.56	− 0.55
	***p* < 0.001**	***p* < 0.001**	***p* < 0.001**
UPDRS swallow score	0.39	0.40	0.42
	***p* = 0.002**	***p* < 0.001**	***p* < 0.001**

Bold printed *p* values represent statistically significant results. Due to multiple testing with 15 tests, results were considered significant in case of *p* < 0.05/15 = 0.003

*PAS* penetration-aspiration scale,* FOIS* functional oral intake scale,* UPDRS *United-Parkinson-Disease-Rating-Scale

### Reliability Analysis

Inter-rater reliability for the overall DIGEST-FEES was Kappa = 0.82, for the subscale efficiency Kappa = 0.80, and for the subscale safety Kappa = 0.83. Intra-rater reliability for the overall DIGEST-FEES was Kappa = 0.87, and for the subscale efficiency Kappa = 0.95. Due to the rare frequency of penetration and aspiration in the random selected subjects, no calculation of intra-rater reliability was possible for the subscale of swallowing safety.

## Discussion

The main finding of this study is that DIGEST-FEES is a reliable and valid patient-based outcome score that can be used to assess the severity of pharyngeal dysphagia in patients with PD. The results of the Delphi survey suggest good content validity for patients with PD. In addition, the survey results indicate, that the score might also be applicable to patients with neurogenic dysphagia in general, although criterion validity was not provided in a general neurological collective in this study. Despite the overall positive evaluation of the score in the expert panel, some points of criticism were also raised, which may represent a starting point for further development and adaptation of the score to PD or neurological collectives in future research. Thus, it was noted that pharyngeal hypesthesia and impaired secretion management may be an important mechanism in neurogenic dysphagia, which is not considered in DIGEST-FEES. Bolus swallow trials may not be possible, particularly in patients with severe dysphagia and impaired secretion management. Therefore, DIGEST-FEES may be less appropriate for this specific group of patients, or alternatively, another procedure may need to be established for how to score patients in whom bolus administration is not possible. In contrast, patients with PD (and possibly also other chronic neurogenic dysphagia) rarely present with secretory retention, but by far the most common dysphagia mechanism is pharyngeal residue [13], for which DIGEST is well suited.

In the graduation of swallowing safety, the dimension of frequency of PAS events was generally evaluated positively by the expert panel. However, the importance of the length of the examination protocol was raised. In this context, it should be taken into account, that FEES protocols often provide 3 swallowing trials for 3 different consistencies, as in the following studies [9, 22, 23, 29, 36–41]. It should be noted that the scenario of the intermittent event (with several, but < 50% of the trials of a single consistency) cannot occur with this protocol specification. Here, either 1/3 of the trials of a consistency are affected (single event) or 2/3 of the trials (chronic). Unlike with penetration and aspiration, the frequency of residue is not a separate dimension in the DIGEST-FEES, but only the maximum residue finding is considered (although a distinction is made between whether residue occurred at only one consistency or at multiple consistencies). In this regard, it was noted in the expert interview that intraindividual variability is considered higher for penetration and aspiration than for pharyngeal residue, which supports the score approach to account the frequency of events when scoring swallowing safety. However, future studies could investigate intraindividual variability of swallowing findings and the influence of different examination protocols (e.g., protocol length) on the DIGEST-FEES results. This would allow empirical investigation of whether the score approach to frequency of findings or considering the maximum finding is appropriate or may require (disease-specific) adjustment.

The experts on the panel also agreed in principle with the procedure for scoring pharyngeal residue. However, it was critically noted that in PD and neurogenic dysphagia, anatomically defined scores (e.g., residue related to cavity filling) rather than bolus-clearance-defined scores are common. In contrast to patients with head and neck cancer, in whom the cavity anatomy may be significantly altered, the pharyngeal cavities often provide important landmarks in assessing the severity of the residue in neurological patients. On the same note, some experts also criticized the defined cut-off values for pharyngeal residue scoring, which deviate from the usual anatomy-based scales such as the Yale-Residue-Scale [34]. In addition, any ordinal subdivision of residue was found to be somewhat arbitrarily set by some of the experts. To overcome this problem, Curtis et al. have previously proposed metric visual analog scales [42]. In future studies, these metric scoring concepts might also be implemented in the further development of the DIGEST-FEES. Whether visual analog scales, as originally envisioned by Curtis et al., are also useful for assessing swallowing safety remains to be investigated. In this context, it seems at least problematic that not all penetration and aspiration events in FEES remain present in the postdeglutitive phase. For example, there are also penetration or aspiration events in which the bolus disappears in the trachea or is removed from the larynx. Therefore, clinically relevant penetration and aspiration events cannot necessarily be classified solely on the basis of the visual analog scale for postdeglutitive residue in the laryngeal vestibule.

In addition, it is important to emphasize that the DIGEST-FEES only classifies the global severity of pharyngeal dysphagia and does not provide information about oral dysfunction or the mechanism of dysphagia. Therefore, mechanistically oriented classifications that characterize, the phenotype of dysphagia [13] or specific oropharyngeal PD mechanisms [22] should be considered as complementary. The same applies to surrogate parameters that allow pathophysiological inferences, such as white-out duration as a parameter for pharyngeal bradykinesia [23] or white-out strength as a parameter for pharyngeal contractility [38].

Besides content validity, our study empirically supported criterion validity by association with common outcome scores. There was clear correlation between the DIGEST-FEES with the Yale-Residue-Scale, with decreased FOIS, and with subjective impairment of swallowing in the UPDRS. However, no association was found between DIGEST-FEES and the PAS. This may be explained by the fact that penetration and aspiration events were rare in our cohort, and thus the DIGEST-FEES was essentially determined by impaired swallowing efficiency. Nevertheless, as expected, there was an association between the PAS and the impaired swallowing efficiency subscore. When examining the magnitude of the correlation coefficients, the greatest agreement was found with the Yale-Valleculae-Residue-Scale, which corresponds to the most common phenotype of PD-related dysphagia with residue in the valleculae [13]. Further, it becomes evident that the relationship between DIGEST-FEES and the analysed outcome parameters was predominantly influenced by the DIGEST efficiency subscore, with the safety subscore indicating partially significant but comparatively weak associations. Consequently, there is a requirement for further investigation to quantify the degree of correlation between the efficiency subscore and other outcome parameters beyond the PAS. Additionally, this research should aim to ascertain whether this correlation corresponds to a clinically significant effect size. Besides criterion validity, the DIGEST-FEES demonstrated good inter-rater and intra-rater reliability.

The results of our study are largely consistent with a VFSS study on 20 PD patients in which the DIGEST was used to investigate associations with dysphagia symptoms and global severity ratings of dysphagia [18]. Consistent with the results of our study, an association between the DIGEST (total score and efficiency subscore) and the FOIS was shown. There was also an association between the global impression of dysphagia impairment according to the overall VFSS examination on a visual analog scale and the DIGEST (total score and both subscores). However, the subjective symptom perception did not show a clear association with the DIGEST. Thus, the sum score of subjective dysphagia symptoms according to the Swallowing Disturbance Questionnaire (SDQ) was not associated with the DIGEST total score. Nevertheless, there was a tendency of an association between the DIGEST-efficiency score and the SDQ (*p* = 0.09). Also consistent with our study results, the authors report high inter-rater and intra-rater reliability for the DIGEST. Thus, this study combined with the results of our study overall suggest good criterion validity and reliability of the DIGEST with VFSS as well as the DIGEST-FEES for PD patients.

There are a number of limitations that must be considered when interpreting the study. Only a relatively small panel of experts participated in the Delphi survey. The cohort consisted of rather mildly affected dysphagic patients, and patients with tube feeding and deep brain stimulation were excluded, so the cohort might not be representative for PD patients in general. In particular, it is important to note that patient populations may vary across different countries and contexts; for instance, deep brain stimulation may not be regarded as an escalation therapy in certain countries and contexts. Therefore, it is important to note that the results of this study may partly vary in other clinical environments. Reliability was determined only in a small sample, and for swallowing safety only some of the ordinal levels of the score were represented. However, reliability has already been studied extensively in the head and neck cancer collective [17], and it seems plausible to assume that reliability may be similar in the neurological collective. In the UPDRS, only two short questions were used as patient-reported outcome measures. Future studies should therefore also examine more detailed and validated patient reported outcome scores.

In summary, the DIGEST-FEES is a reliable and valid score for quantifying global pharyngeal dysphagia severity at the patient level in PD patients. Nevertheless, there are individual domains that should possibly be modified and adapted to PD and the neurological collective in the future to further optimize the score.

### Supplementary Information

Below is the link to the electronic supplementary material.

Supplementary file1 (DOCX 20 kb)

Supplementary file2 (DOCX 1702 kb)

Supplementary file3 (DOCX 1664 kb)

## Data Availability

The data of the Delphi survey can be found in supplementary material 2 and 3. All further data are restricted by the ethics vote due to data protection and are not publicly available.
