# Supplementary material for: Validation of the DIGEST-FEES as a Global Outcome Measure for Pharyngeal Dysphagia in Parkinson’s Disease
Source: Dysphagia. 2023 Dec 22;39(4):697–704. doi: 10.1007/s00455-023-10650-6 (PMC11239722; doi:10.1007/s00455-023-10650-6)
Supplement: Supplementary file 1 — Supplementary file1 (DOCX 20 kb) [file 455_2023_10650_MOESM1_ESM.docx]

**Content validity survey DIGEST-FEES:**

1.1 The DIGEST-FEES grades the severity of pharyngeal dysphagia according to impairment of swallowing safety and impairment in efficiency of bolus clearance. Are these also parameters that comprehensively characterize clinically relevant pharyngeal dysphagia in patients with Parkinson's disease?

| Strongly agree | agree | Neither agree nor disagree | Disagree | Strongly disagree |
| --- | --- | --- | --- | --- |

If you do not fully agree or have anything else to comment on, you can do so here.

1.2 Are these also parameters that comprehensively characterize neurogenic pharyngeal dysphagia?

| Strongly agree | agree | Neither agree nor disagree | Disagree | Strongly disagree |
| --- | --- | --- | --- | --- |

If you do not fully agree or have anything else to comment on, you can do so here.

2.1 The DIGEST-FEES uses the maximum Penetration Aspiration Scale according to Rosenbek during the examination as basis for the classification of swallowing safety. Is this approach in principle also suitable for patients with Parkinson's disease?

| Strongly agree | agree | Neither agree nor disagree | Disagree | Strongly disagree |
| --- | --- | --- | --- | --- |

If you do not fully agree or have anything else to comment on, you can do so here.

2.2 Is this approach in principle also suitable for neurogenic dysphagia?

| Strongly agree | agree | Neither agree nor disagree | Disagree | Strongly disagree |
| --- | --- | --- | --- | --- |

If you do not fully agree or have anything else to comment on, you can do so here.

3.1 When determining swallowing safety, the DIGEST-FEES divides into PAS 1/2 (no penetration/aspiration or flash penetration above the true vocal folds), PAS 3/4 (silent penetration above true vocal folds or flash penetration to the vocal folds), PAS 5/6 (silent penetration to the true vocal folds or flash aspiration) and PAS 7/8 (aspiration not cleared, silent or sensate). Is this classification also suitable for Parkinson's patients?

| Strongly agree | agree | Neither agree nor disagree | Disagree | Strongly disagree |
| --- | --- | --- | --- | --- |

If you do not fully agree or have anything else to comment on, you can do so here.

3.2 Is this classification also suitable for neurological patients?

| Strongly agree | agree | Neither agree nor disagree | Disagree | Strongly disagree |
| --- | --- | --- | --- | --- |

If you do not fully agree or have anything else to comment on, you can do so here.

4.1 The DIGEST-FEES also takes into account whether a penetration or aspiration event occurred only once, whether it occurred intermittently (on multiple but > 50% of trials on a single consistency), or whether it occurred chronically (majority of thin liquid trials and/or on > 1 consistency). Is this distinction also useful in patients with Parkinson's disease?

| Strongly agree | agree | Neither agree nor disagree | Disagree | Strongly disagree |
| --- | --- | --- | --- | --- |

If you do not fully agree or have anything else to comment on, you can do so here.

4.2 Is this distinction also useful in neurogenic pharyngeal dysphagia?

| Strongly agree | agree | Neither agree nor disagree | Disagree | Strongly disagree |
| --- | --- | --- | --- | --- |

If you do not fully agree or have anything else to comment on, you can do so here.

5.1 For penetration or aspiration events of ≥5, the DIGEST-FEES distinguishes between gross events (>25% of bolus volume) and non-gross events. Is this distinction also useful in patients with Parkinson's disease?

| Strongly agree | agree | Neither agree nor disagree | Disagree | Strongly disagree |
| --- | --- | --- | --- | --- |

If you do not fully agree or have anything else to comment on, you can do so here.

5.2 Is this distinction also useful in neurogenic pharyngeal dysphagia?

| Strongly agree | agree | Neither agree nor disagree | Disagree | Strongly disagree |
| --- | --- | --- | --- | --- |

If you do not fully agree or have anything else to comment on, you can do so here.

6.1 Is the categorization of impaired swallowing safety in the DIGEST-FEES overall useful for patients with Parkinson's disease?

| Strongly agree | agree | Neither agree nor disagree | Disagree | Strongly disagree |
| --- | --- | --- | --- | --- |

If you do not fully agree or have anything else to comment on, you can do so here.

6.2 Is the categorization of impaired swallowing safety in the DIGEST-FEES overall useful for pharyngal neurogenic dysphagia?

| Strongly agree | agree | Neither agree nor disagree | Disagree | Strongly disagree |
| --- | --- | --- | --- | --- |

If you do not fully agree or have anything else to comment on, you can do so here.

7.1 The DIGEST-FEES efficiency grade considers the maximum percentage of residue in the pharynx, taking into account the size of the bolus administered and the amount of residue in the overall pharynx ("overall, how much residue do you see?"). Is this approach also useful for patients with Parkinson's disease?

| Strongly agree | agree | Neither agree nor disagree | Disagree | Strongly disagree |
| --- | --- | --- | --- | --- |

If you do not fully agree or have anything else to comment on, you can do so here.

7.2 Is this approach also useful for patients with neurogenic pharyngeal dysphagia?

| Strongly agree | agree | Neither agree nor disagree | Disagree | Strongly disagree |
| --- | --- | --- | --- | --- |

If you do not fully agree or have anything else to comment on, you can do so here.

8.1 The DIGEST-FEES evaluates pharyngeal residue of less than 10% ("minimal to no residue") as clinically not relevant. Is this also appropriate for patients with Parkinson's disease?

| Strongly agree | agree | Neither agree nor disagree | Disagree | Strongly disagree |
| --- | --- | --- | --- | --- |

If you do not fully agree or have anything else to comment on, you can do so here.

8.2 Is this also appropriate for patients with pharyngeal neurogenic dysphagia?

| Strongly agree | agree | Neither agree nor disagree | Disagree | Strongly disagree |
| --- | --- | --- | --- | --- |

If you do not fully agree or have anything else to comment on, you can do so here.

9.1 The DIGEST-FEES evaluates pharyngeal residue of 10%-33% as mild impairment of swallowing efficiency. Is this also appropriate for patients with Parkinson's disease?

| Strongly agree | agree | Neither agree nor disagree | Disagree | Strongly disagree |
| --- | --- | --- | --- | --- |

If you do not fully agree or have anything else to comment on, you can do so here.

9.2 Is this also appropriate for patients with pharyngeal neurogenic dysphagia?

| Strongly agree | agree | Neither agree nor disagree | Disagree | Strongly disagree |
| --- | --- | --- | --- | --- |

If you do not fully agree or have anything else to comment on, you can do so here.

10.1 The DIGEST-FEES evaluates pharyngeal residue of 34%-66% ("majority residue") as moderate impairment of swallowing efficiency. Is this also appropriate for patients with Parkinson's disease?

| Strongly agree | agree | Neither agree nor disagree | Disagree | Strongly disagree |
| --- | --- | --- | --- | --- |

If you do not fully agree or have anything else to comment on, you can do so here.

10.2 Is this also appropriate for patients with pharyngeal neurogenic dysphagia?

| Strongly agree | agree | Neither agree nor disagree | Disagree | Strongly disagree |
| --- | --- | --- | --- | --- |

If you do not fully agree or have anything else to comment on, you can do so here.

11.1 The DIGEST-FEES evaluates pharyngeal residue of greater than 66% ("nearly complete residue") as severe impairment of swallowing efficiency. Is this also appropriate for patients with Parkinson's disease?

| Strongly agree | agree | Neither agree nor disagree | Disagree | Strongly disagree |
| --- | --- | --- | --- | --- |

If you do not fully agree or have anything else to comment on, you can do so here.

11.2 Is this also appropriate for patients with pharyngeal neurogenic dysphagia?

| Strongly agree | agree | Neither agree nor disagree | Disagree | Strongly disagree |
| --- | --- | --- | --- | --- |

If you do not fully agree or have anything else to comment on, you can do so here.

12.1 For moderate residue, the DIGEST-FEES assigns a higher impairment grade if the residue occurs on liquid and semisolid consistency swallowing trails, rather than only in solid consistency swallowing trails. Is this approach also appropriate for patients with Parkinson's disease?

| Strongly agree | agree | Neither agree nor disagree | Disagree | Strongly disagree |
| --- | --- | --- | --- | --- |

If you do not fully agree or have anything else to comment on, you can do so here.

12.2 Is this approach also appropriate for patients with pharyngeal neurogenic dysphagia?

| Strongly agree | agree | Neither agree nor disagree | Disagree | Strongly disagree |
| --- | --- | --- | --- | --- |

If you do not fully agree or have anything else to comment on, you can do so here.

13.1 For severe residue, the DIGEST-FEES assigns a higher level of impairment if the residue occurred on all bolus types presented, rather than just any, but not all bolus types. Is this approach also suitable for patients with Parkinson's disease?

| Strongly agree | agree | Neither agree nor disagree | Disagree | Strongly disagree |
| --- | --- | --- | --- | --- |

If you do not fully agree or have anything else to comment on, you can do so here.

13.2 Is this approach also appropriate for patients with pharyngeal neurogenic dysphagia?

| Strongly agree | agree | Neither agree nor disagree | Disagree | Strongly disagree |
| --- | --- | --- | --- | --- |

If you do not fully agree or have anything else to comment on, you can do so here.

14.1 Is the classification of impaired swallowing efficiency overall also appropriate for patients with Parkinson's disease?

| Strongly agree | agree | Neither agree nor disagree | Disagree | Strongly disagree |
| --- | --- | --- | --- | --- |

If you do not fully agree or have anything else to comment on, you can do so here.

14.2 Is the categorization of impaired swallowing efficiency overall also useful for pharyngeal neurogenic dysphagia?

| Strongly agree | agree | Neither agree nor disagree | Disagree | Strongly disagree |
| --- | --- | --- | --- | --- |

If you do not fully agree or have anything else to comment on, you can do so here.

15.1 Is the intended interaction between swallowing safety and swallowing efficiency in determining the overall impairment level in the DIGEST-FEES appropriate in patients with Parkinson's disease?

| Strongly agree | agree | Neither agree nor disagree | Disagree | Strongly disagree |
| --- | --- | --- | --- | --- |

If you do not fully agree or have anything else to comment on, you can do so here.

15.2 Is the intended interaction between swallowing safety and swallowing efficiency in determining the overall impairment level in the DIGEST-FEES also useful in patients with pharyngeal neurogenic dysphagia?

| Strongly agree | agree | Neither agree nor disagree | Disagree | Strongly disagree |
| --- | --- | --- | --- | --- |

If you do not fully agree or have anything else to comment on, you can do so here.
