# Supplementary material for: Validation of the DIGEST-FEES as a Global Outcome Measure for Pharyngeal Dysphagia in Parkinson’s Disease
Source: Dysphagia. 2023 Dec 22;39(4):697–704. doi: 10.1007/s00455-023-10650-6 (PMC11239722; doi:10.1007/s00455-023-10650-6)
Supplement: Supplementary file 2 — Supplementary file2 (DOCX 1702 kb) [file 455_2023_10650_MOESM2_ESM.docx]

**Expert Survey DIGEST-FEES: Results first Delphi-Round**

- 1. The DIGEST-FEES grades the severity of pharyngeal dysphagia according to impairment of swallowing safety and impairment in efficiency of bolus clearance. Are these parameters that comprehensively characterize clinically relevant pharyngeal dysphagia in patients with Parkinson's disease?


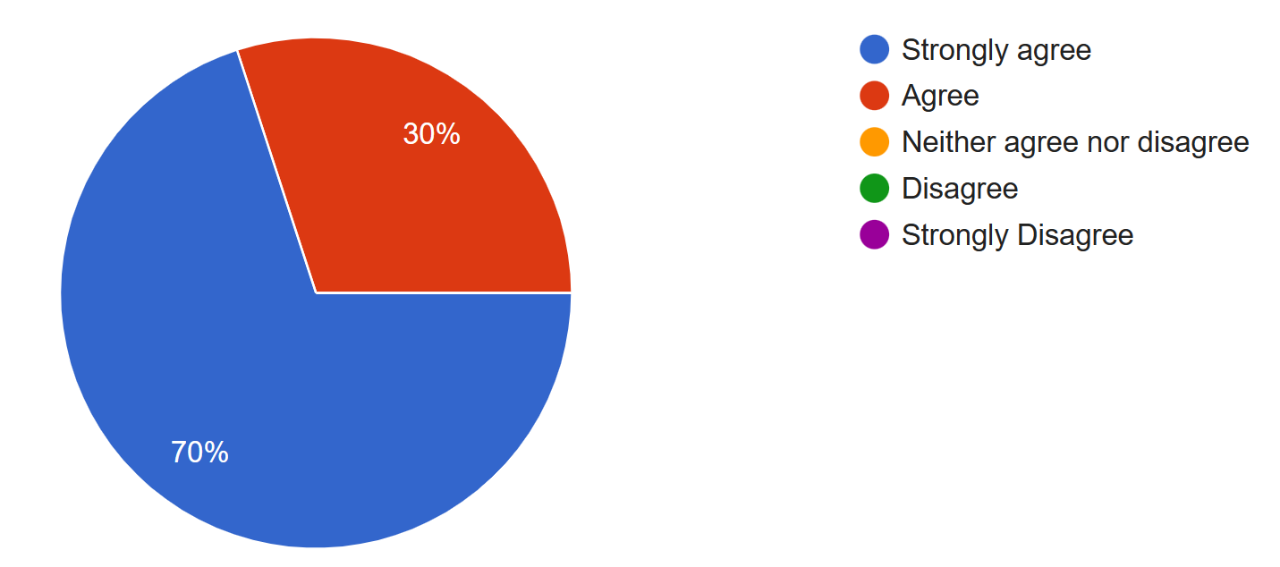


1.2 If you do not fully agree or have anything else to comment on, you can do so here.

- Premature bolus spillage represents another dysfunction that may be noted in the pharyngeal phase but is formally an oral dysfunction. Therefore, I agree, but it is important to note that the classification only applies to the pharyngeal phase and not to the oropahryngeal phase. On the other hand, premature bolus spillage is mostly only relevant, if this leads to penetration or aspiration, which is captured in the score.
- PD dysphagia does not only comprise pen/asp and pharyngeal residue; these findings are part of it though, and to depict those symptoms, the DIGEST is a valuable tool.
  1. Are these parameters that comprehensively characterize neurogenic pharyngeal dysphagia?


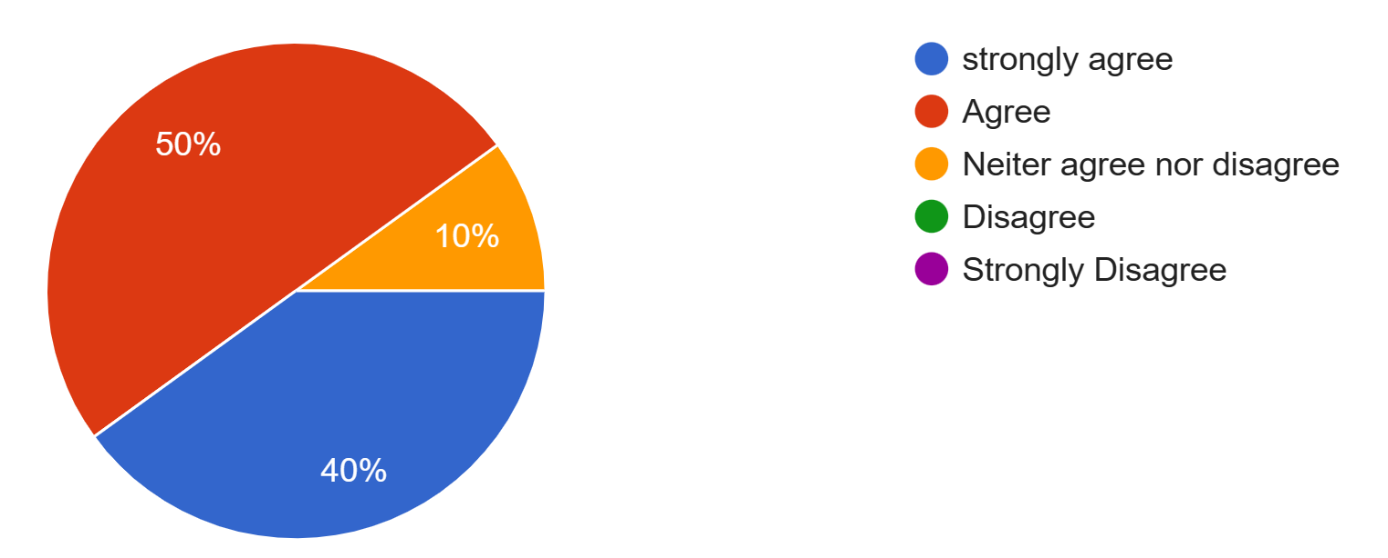


- 1. If you do not fully agree or have anything else to comment on, you can do so here.
     - Premature bolus spillage represents another dysfunction that may be noted in the pharyngeal phase but is formally an oral dysfunction. Therefore, I agree, but it is important to note that the classification only applies to the pharyngeal phase and not to the oropahryngeal phase. On the other hand, premature bolus spillage is mostly only relevant, if this leads to penetration or aspiration, which is captured in the score.
     - In some neurological diseases (such as stroke patients and intensive care patients), secretion management also plays a decisive role, especially in patients with severe dysphagia. Often, in these patients, no testing with several swallowing trails and different bolus consistencies is possible. For these patients, the score in the current version is probably less suitable. For all other patients (especially with chronic dysphagia), I believe the above parameters are appropriate to comprehensively characterize dysphagia.
- Neurogenic dysphagia may comprise of sensory impairment as well which is not displayed here.
- It would be interesting to know what there are pharyngeal dysphagia: due to postdeglutitive leaking, insufficient pharyngeal squeeze, hence pharyngeal bradykinesia, postdeglutitive regurgitation.

2.1 The DIGEST-FEES uses the maximum Penetration Aspiration Scale according to Rosenbek during the examination as basis for the classification of swallowing safety. Is this approach in principle also suitable for patients with Parkinson's disease?


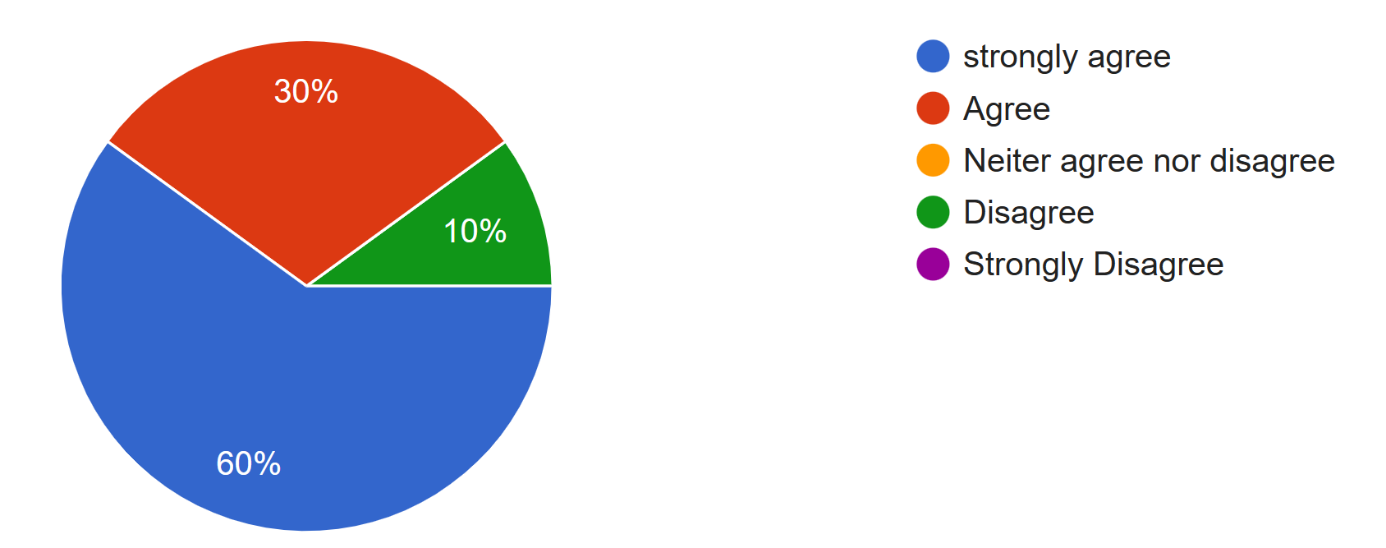


2.2 If you do not fully agree or have anything else to comment on, you can do so here.

- In principle, the PAS has been intensively studied in this cohort of patients and could be associated with important outcome parameters. Nevertheless, in my opinion, the score has some weaknesses. For example, it is not obvious why a PAS of 4 (penetration with touching the vocal folds and clearing) should be worse than a PAS of 3 (penetration without touching the vocal folds, but also without clearing). Especially in FEES, the triggering of protective reflexes (instead of just clearing) could also be taken more into account in the assessment of penetration and aspiration. Nevertheless, from my point of view, there is currently no better validated score than the PAS, so I basically agree with the statement.

2.3 Is this approach in principle also suitable for neurogenic dysphagia?


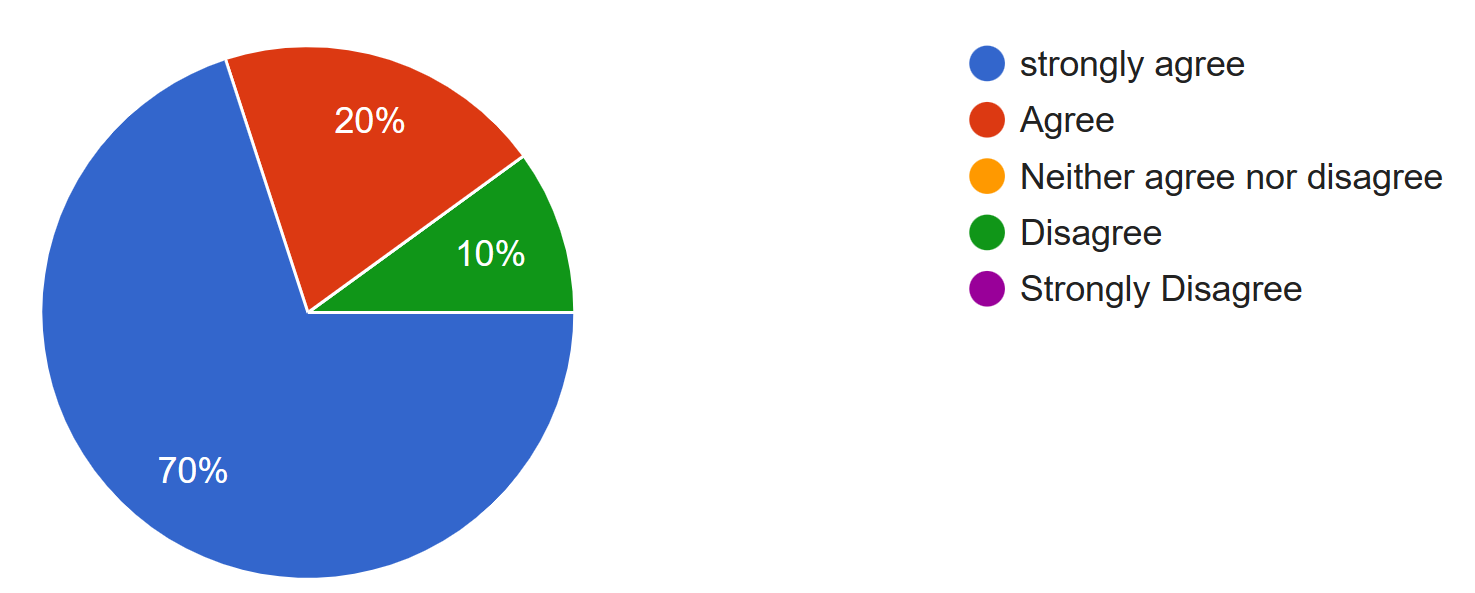


2.4 If you do not fully agree or have anything else to comment on, you can do so here.

- In principle, the PAS has been intensively studied in this cohort of patients and could be associated with important outcome parameters. Nevertheless, in my opinion, the score has some weaknesses. For example, it is not obvious why a PAS of 4 (penetration with touching the vocal folds and clearing) should be worse than a PAS of 3 (penetration without touching the vocal folds, but also without clearing). Especially in FEES, the triggering of protective reflexes (instead of just clearing) could also be taken more into account in the assessment of penetration and aspiration. Nevertheless, from my point of view, there is currently no better validated score than the PAS, so I basically agree with the statement.

3.1 When determining swallowing safety, the DIGEST-FEES divides into PAS 1/2 (no penetration/aspiration or flash penetration above the true vocal folds), PAS 3/4 (silent penetration above true vocal folds or flash penetration to the vocal folds), PAS 5/6 (silent penetration to the true vocal folds or flash aspiration) and PAS 7/8 (aspiration not cleared, silent or sensate). Is this classification also suitable for Parkinson's disease?


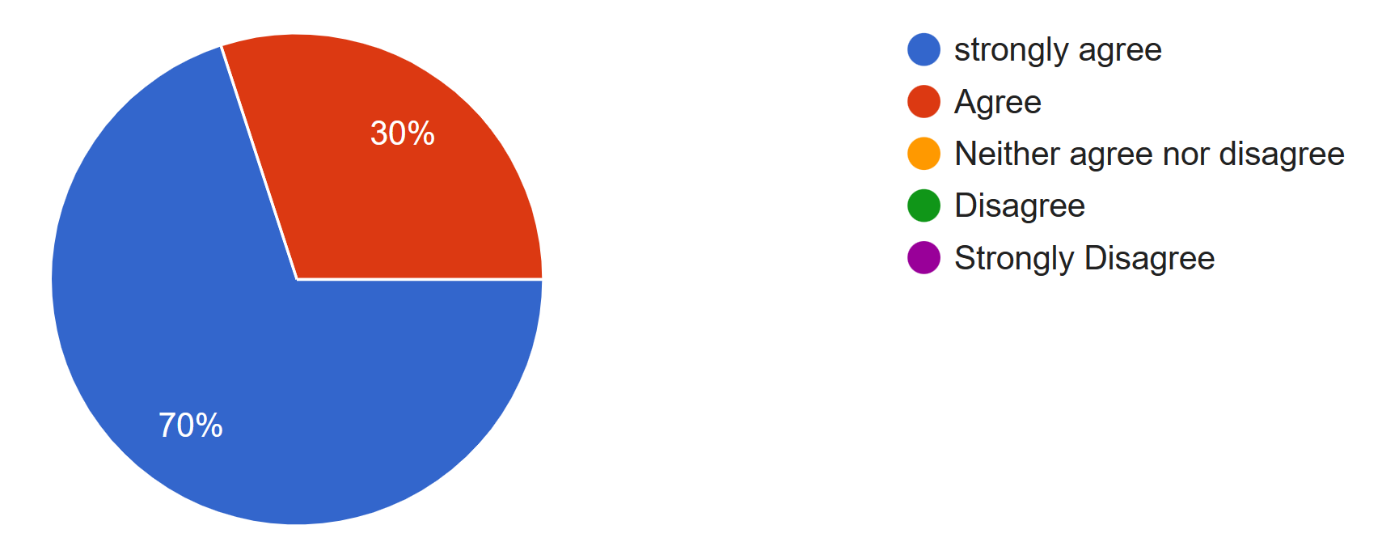


3.2 If you do not fully agree or have anything else to comment on, you can do so here.

- No comments were made here.

3.3 Is this classification also suitable for neurological patients?


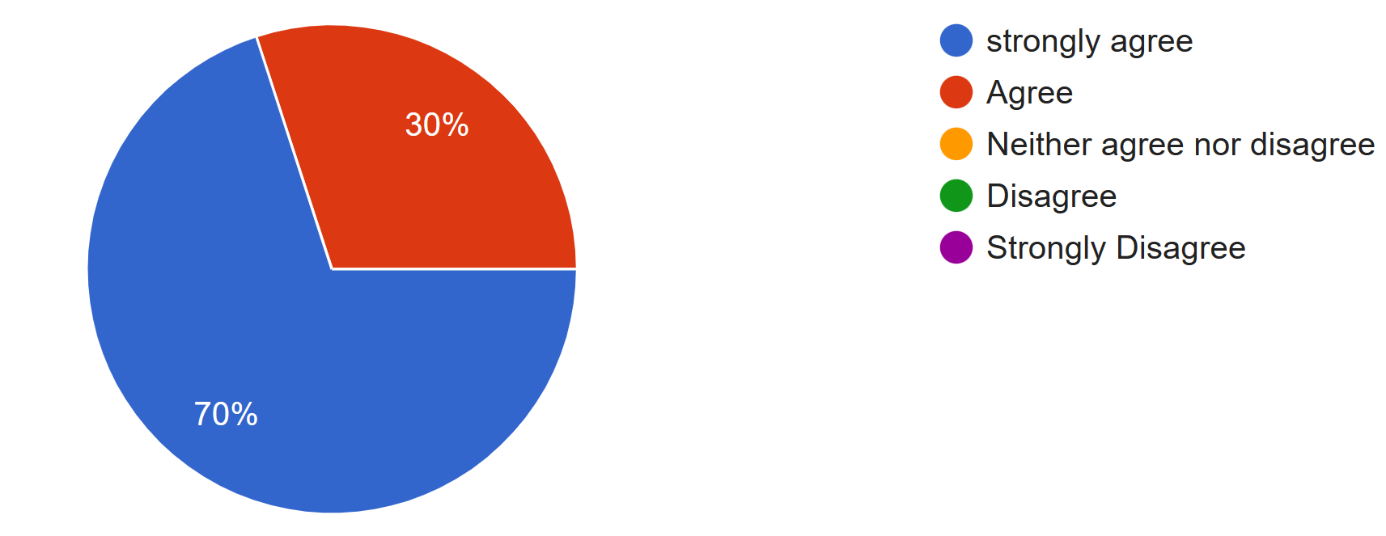


3.4 If you do not fully agree or have anything else to comment on, you can do so here.

- No comments were made here.

4.1 The DIGEST-FEES also takes into account whether a penetration or aspiration event occurred only once, whether it occurred intermittently (on multiple but > 50% of trials on a single consistency), or whether it occurred chronically (majority of thin liquid trials and/or on > 1 consistency). Is this distinction also useful in patients with Parkinson's disease?


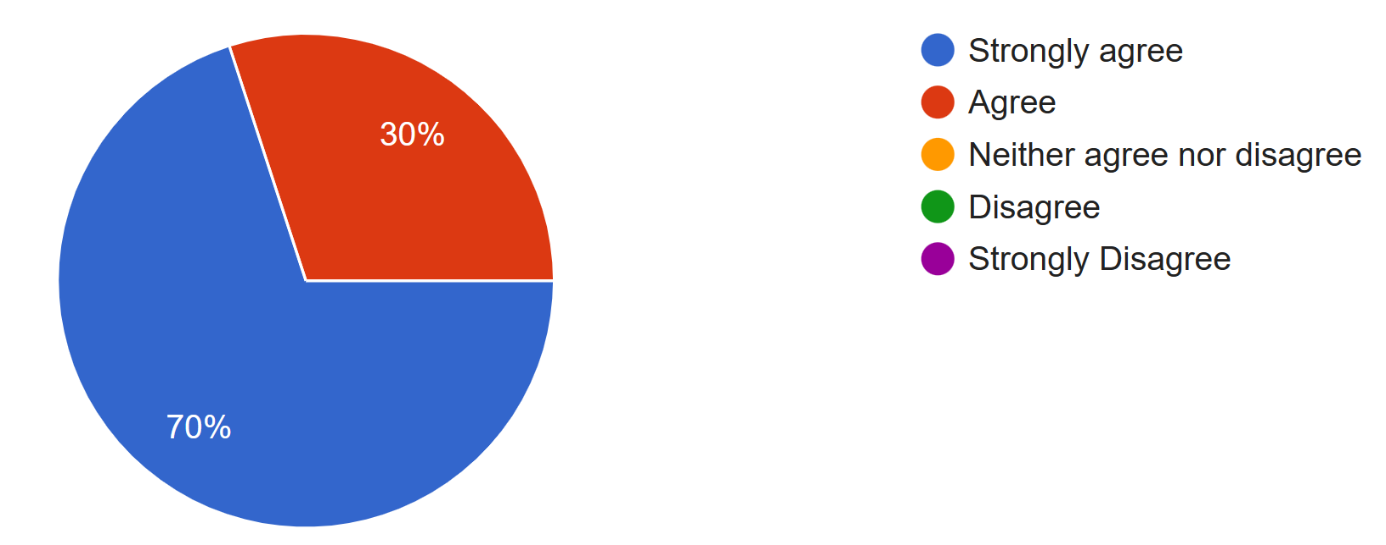


4.2 If you do not fully agree or have anything else to comment on, you can do so here.

- In principle, I agree, but it should be noted that many FEES protocols only provide for 3 swallows per consistency. Thus, the scenario "multiple swallows on <50% of trails on a single consistency) will not occur when using such protocols, so that only a subdivision into single event and chronic is possible there.
- PAS of 8 does not occur only once.

4.3 Is this distinction also useful in neurogenic pharyngeal dysphagia?


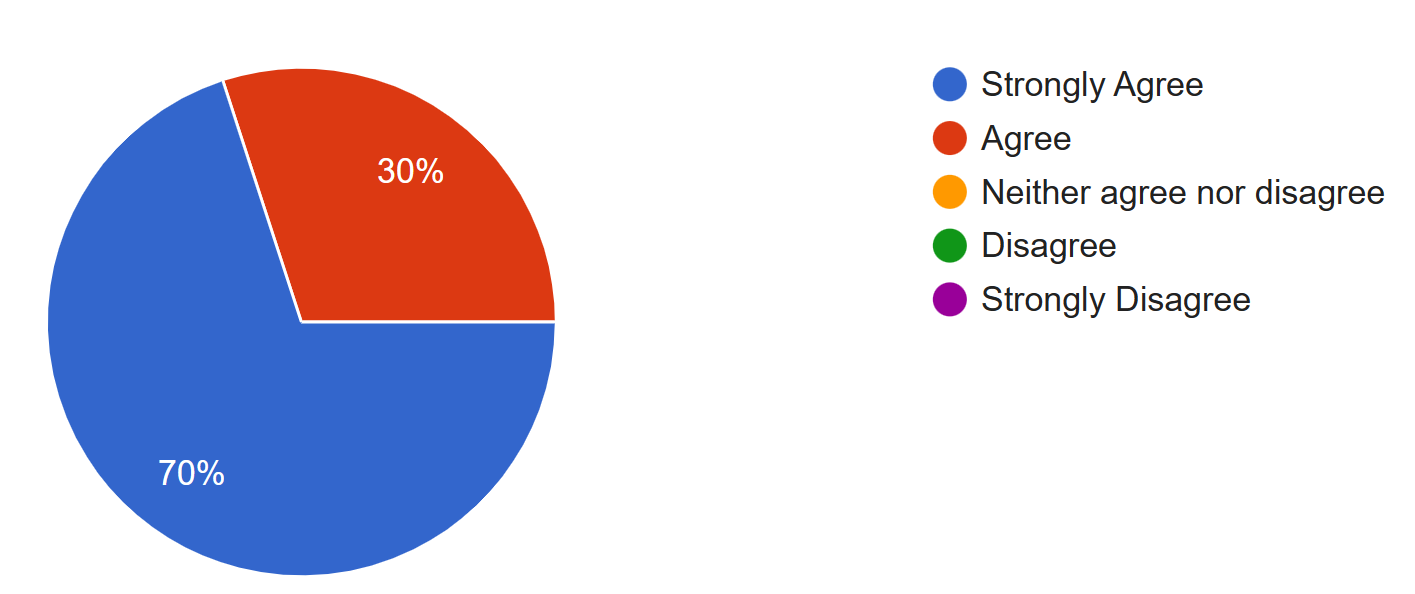


4.4 If you do not fully agree or have anything else to comment on, you can do so here.

- In principle, I agree, but it should be noted that many FEES protocols only provide for 3 swallows per consistency. Thus, the scenario "multiple swallows on <50% of trails on a single consistency) will not occur when using such protocols, so that only a subdivision into single event and chronic is possible there.
- PAS of 8 does not occur only once.

5.1 For penetration or aspiration events of ≥5, the DIGEST-FEES distinguishes between gross events (>25% of bolus volume) and non-gross events. Is this distinction also useful in patients with Parkinson's disease?


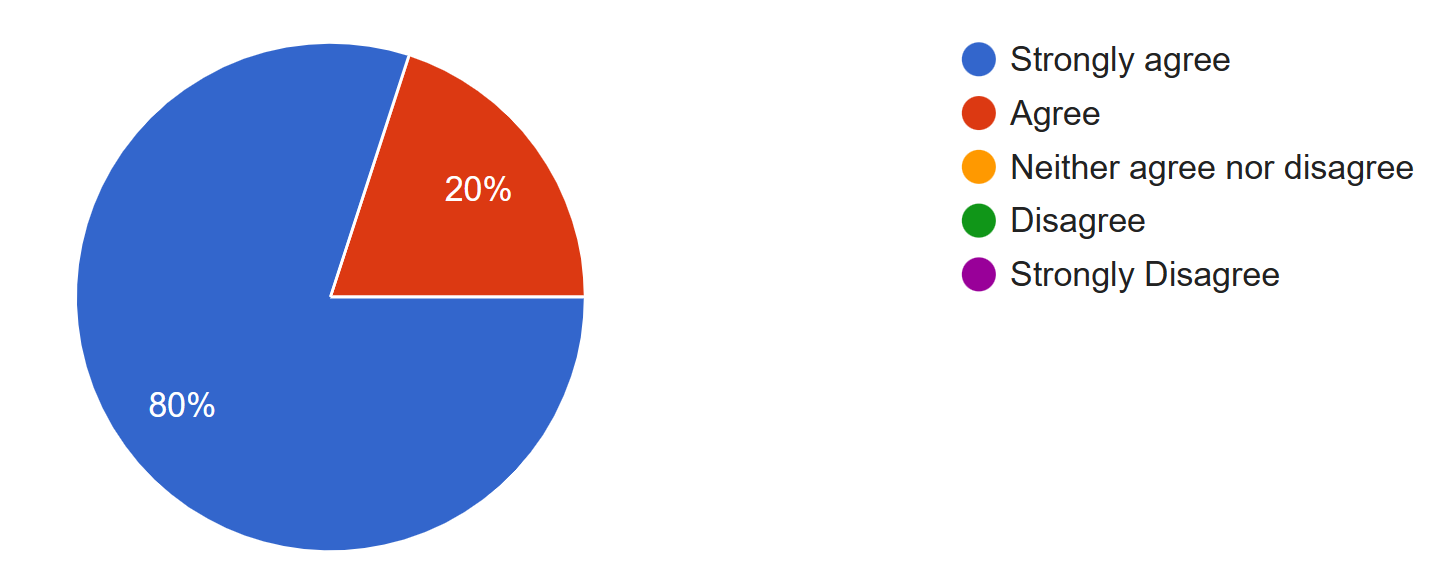


5.2 If you do not fully agree or have anything else to comment on, you can do so here.

- No comments were made here.

5.3 Is this distinction also useful in neurogenic pharyngeal dysphagia?


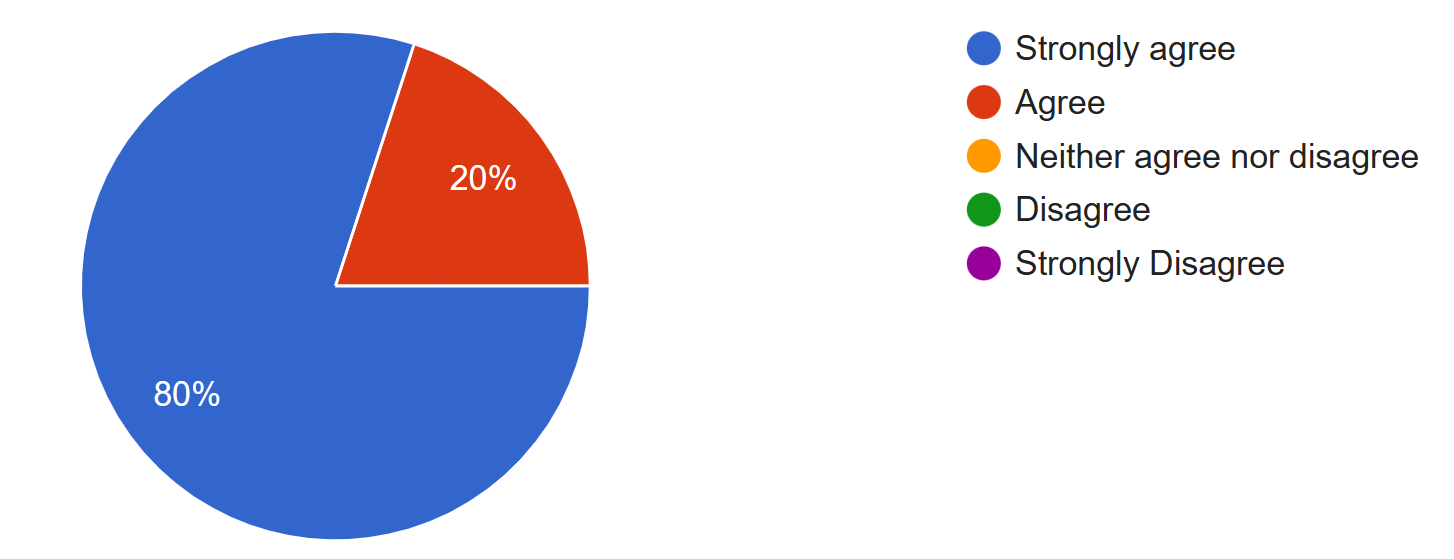


5.4 If you do not fully agree or have anything else to comment on, you can do so here.

- No comments were made here.

6.1 Is the categorization of impaired swallowing safety in the DIGEST-FEES overall useful for patients with Parkinson's disease?


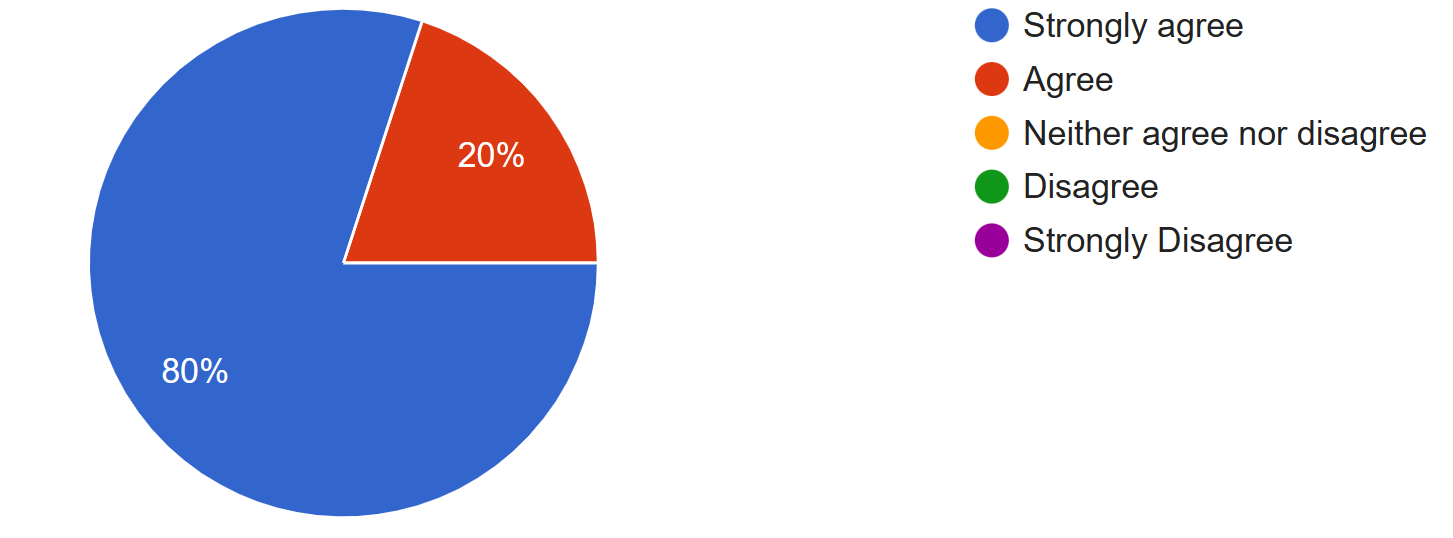


6.2 If you do not fully agree or have anything else to comment on, you can do so here.

- No comments were made here.

6.3 Is the categorization of impaired swallowing safety in the DIGEST-FEES overall useful for pharyngal neurogenic dysphagia?


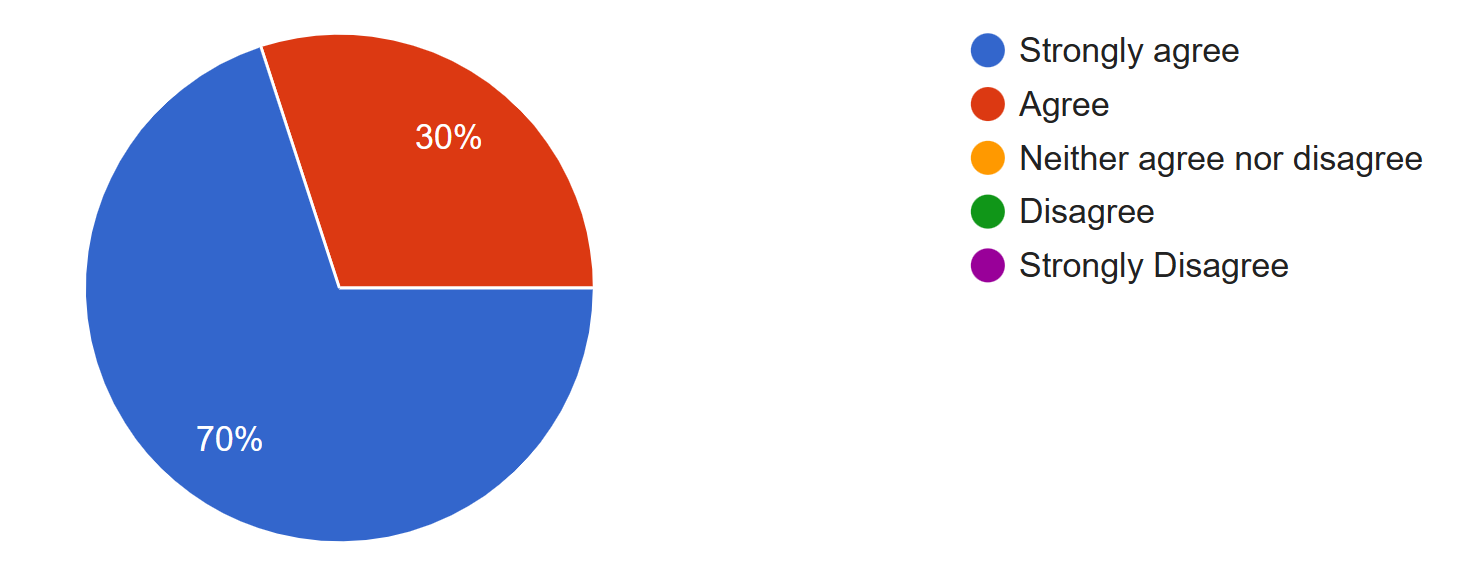


6.4 If you do not fully agree or have anything else to comment on, you can do so here.

- No comments were made here.

7.1 The DIGEST-FEES efficiency grade considers the maximum percentage of residue in the pharynx, taking into account the size of the bolus administered and the amount of residue in the overall pharynx ("overall, how much residue do you see?"). Is this approach also useful for patients with Parkinson's disease?


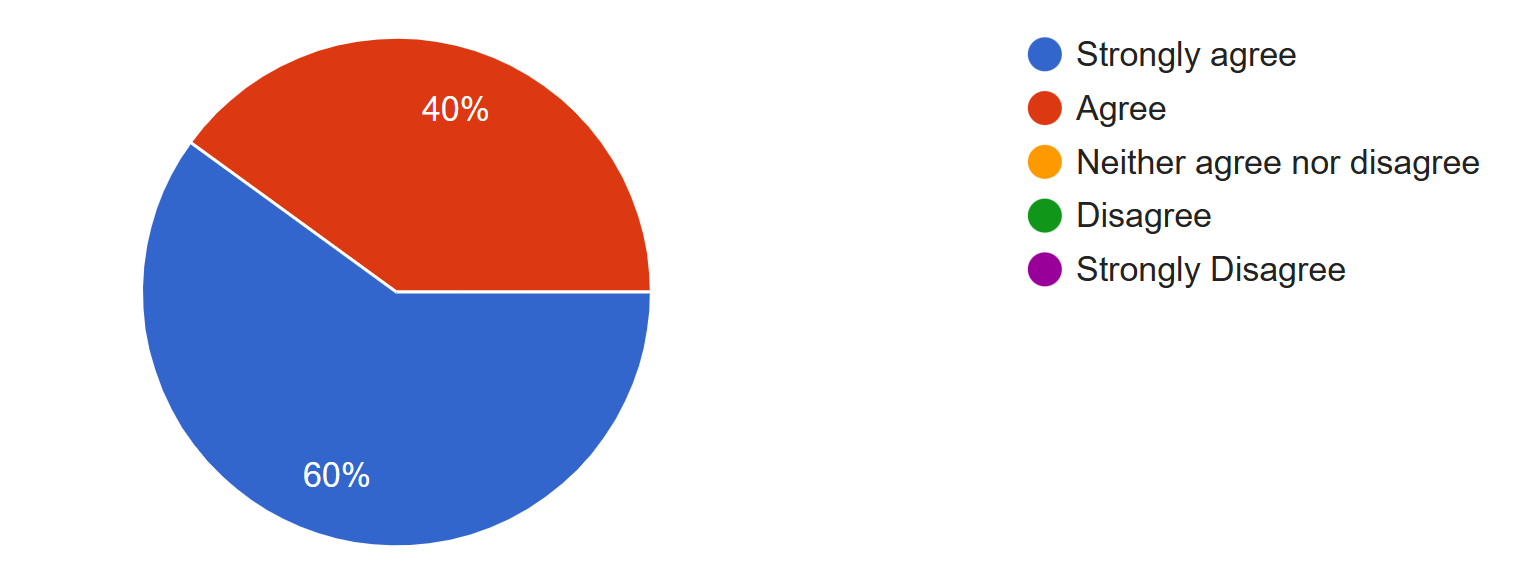


7.2 If you do not fully agree or have anything else to comment on, you can do so here.

- In principle, anatomy-based assessment of residue (i.e., proportion of residue in relation to the pharyngeal recesses) rather than assessment based on bolus clearance is also possible in neurological patients and possibly even superior (in contrast to ENT patients with frequent anatomical changes of the pharyngeal recesses).
- Taking into account the bolus size does indeed make sense but will make clinical applicability more difficult.

7.3 Is this approach also useful for patients with neurogenic pharyngeal dysphagia?


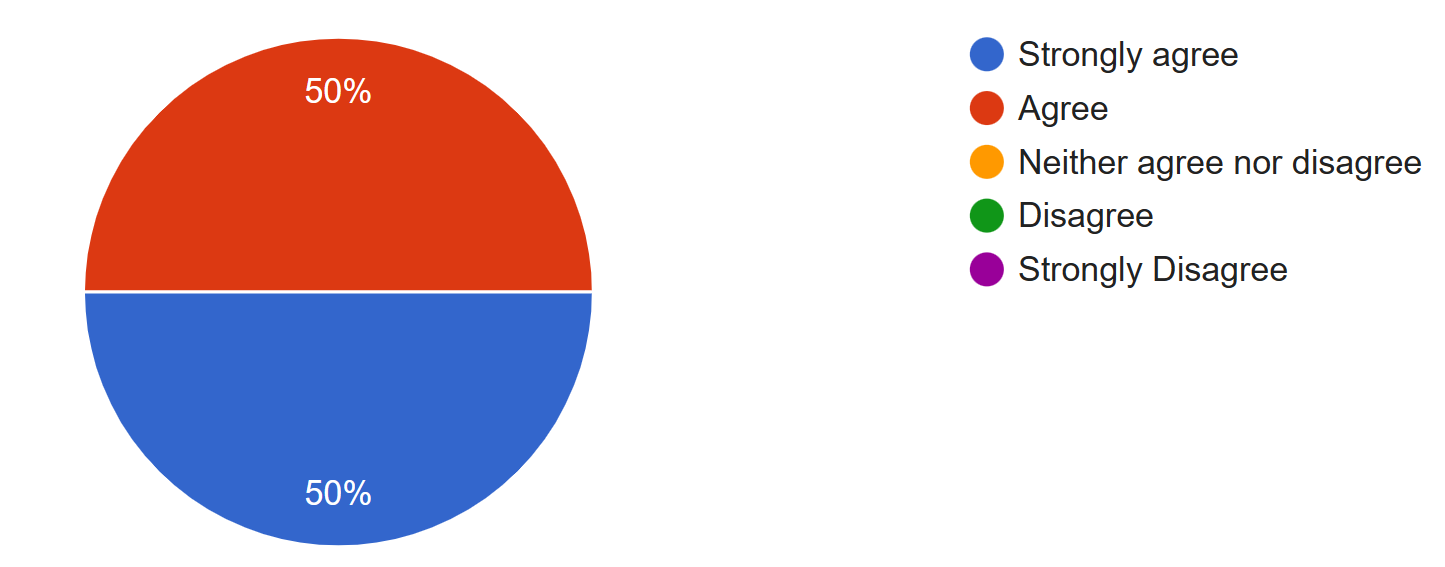


7.4 If you do not fully agree or have anything else to comment on, you can do so here.

- In principle, anatomy-based assessment of residue (i.e., proportion of residue in relation to the pharyngeal recesses) rather than assessment based on bolus clearance is also possible in neurological patients and possibly even superior (in contrast to ENT patients with frequent anatomical changes of the pharyngeal recesses).

8.1 The DIGEST-FEES evaluates pharyngeal residue of less than 10% ("minimal to no residue") as clinically not relevant. Is this also appropriate for patients with Parkinson's disease?


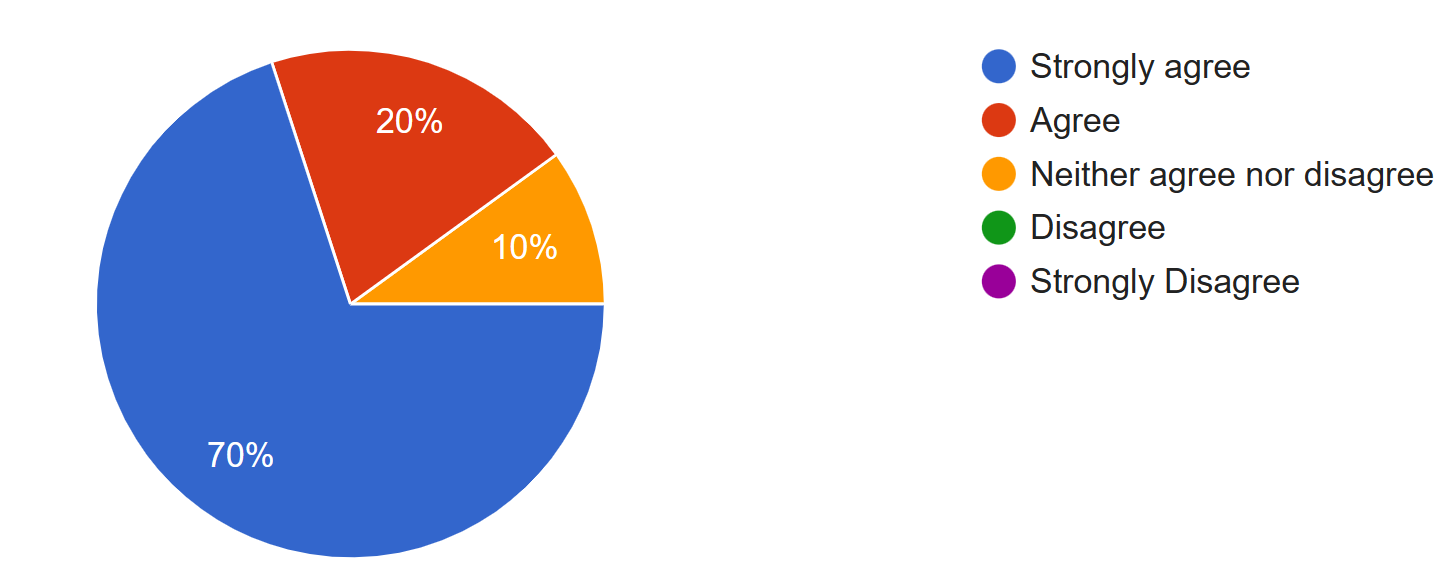


8.2 If you do not fully agree or have anything else to comment on, you can do so here.

- You can only grade the amount, not the clinical relevance. It depends on the protective mechanisms the patient has in place. Usually, PD patients have reduced sensory perception in the larynx, and it then depends on the efficacy of tracheal clearing whether 10% residue might pose a risk to them or not. I suggest attributing minimal residue.

8.3 Is this also appropriate for patients with pharyngeal neurogenic dysphagia?


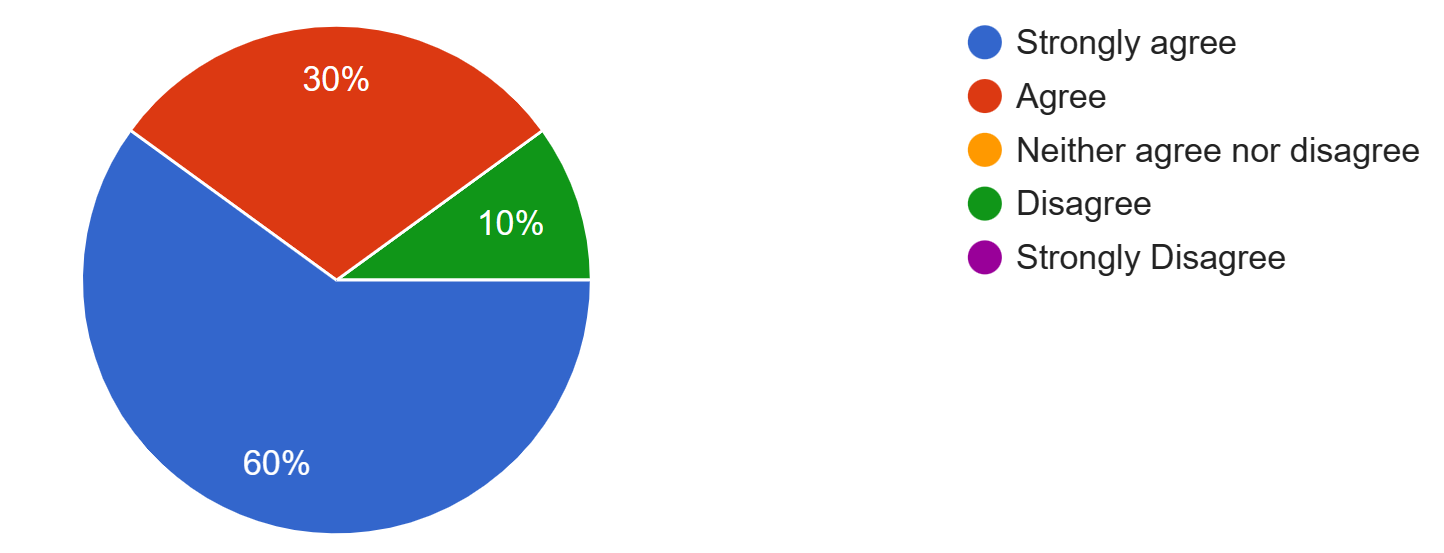


8.4 If you do not fully agree or have anything else to comment on, you can do so here.

- See above.

9.1 The DIGEST-FEES evaluates pharyngeal residue of 10%-33% as mild impairment of swallowing efficiency. Is this also appropriate for patients with Parkinson's disease?


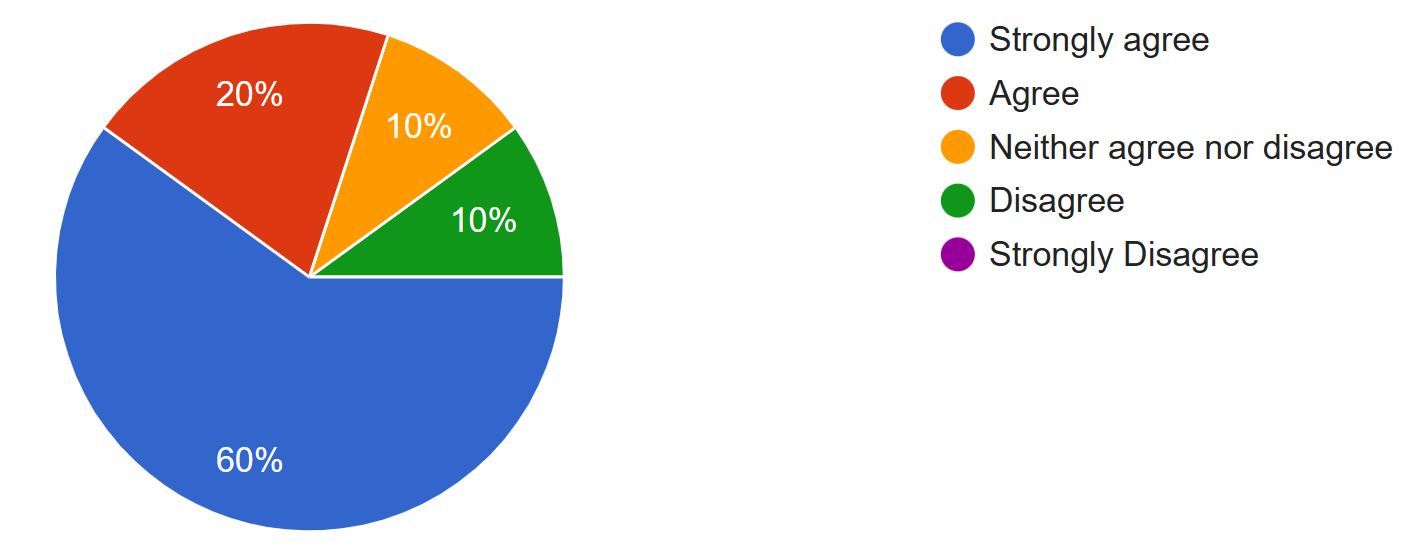


9.2 If you do not fully agree or have anything else to comment on, you can do so here.

- I am not too happy about different percentages between the different residue scores. Why not use 5-25% and 25-50%? These differences make it more academic but - in my opinion - will not lead to a better evaluation in the clinical context.

9.3 Is this also appropriate for patients with pharyngeal neurogenic dysphagia?


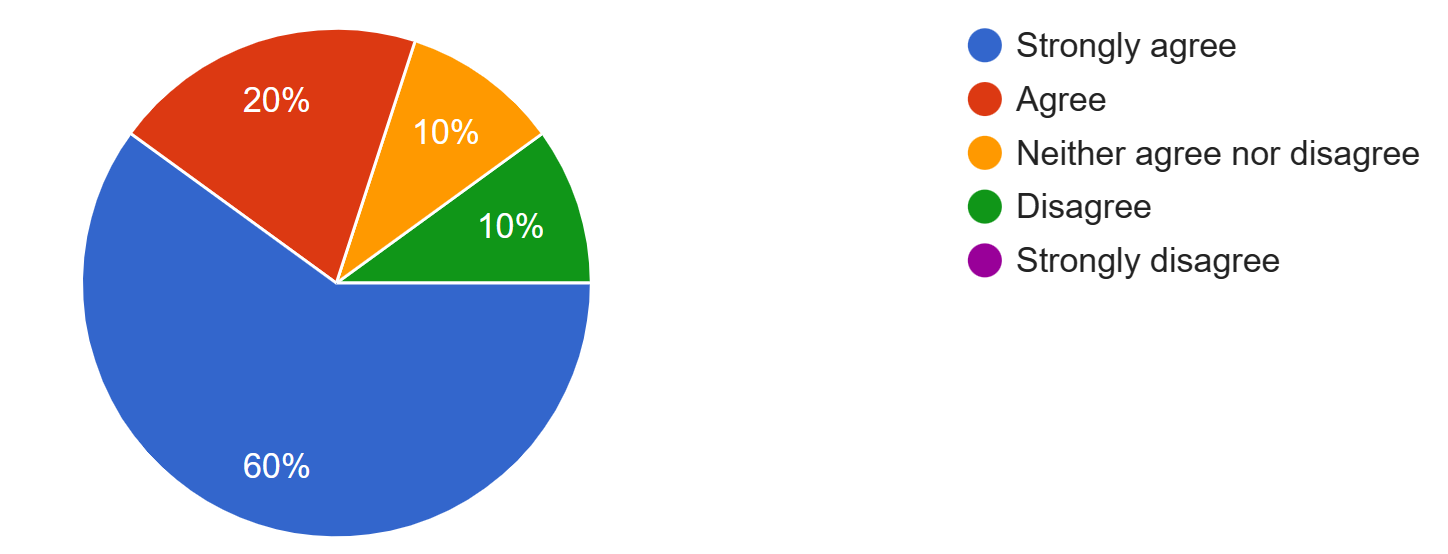


9.4 If you do not fully agree or have anything else to comment on, you can do so here.

- see comment above.

10.1 The DIGEST-FEES evaluates pharyngeal residue of 34%-66% ("majority residue") as moderate impairment of swallowing efficiency. Is this also appropriate for patients with Parkinson's disease?


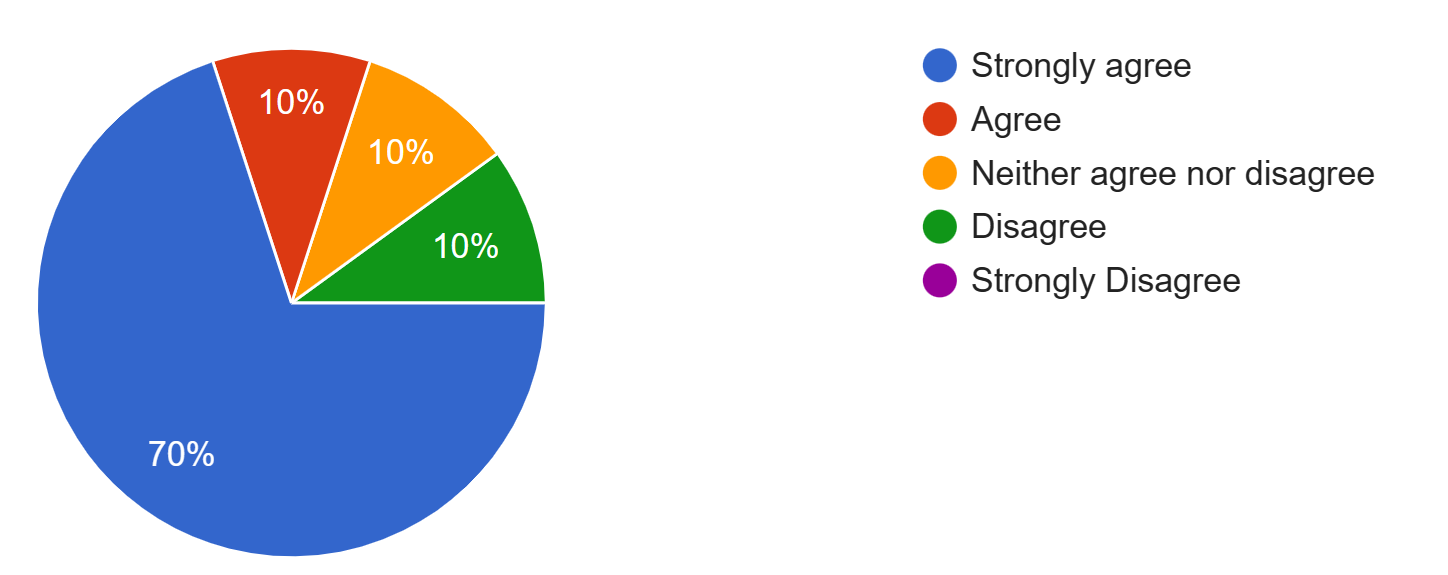


10.2 If you do not fully agree or have anything else to comment on, you can do so here.

- see comment above.

10.3 Is this also appropriate for patients with pharyngeal neurogenic dysphagia?


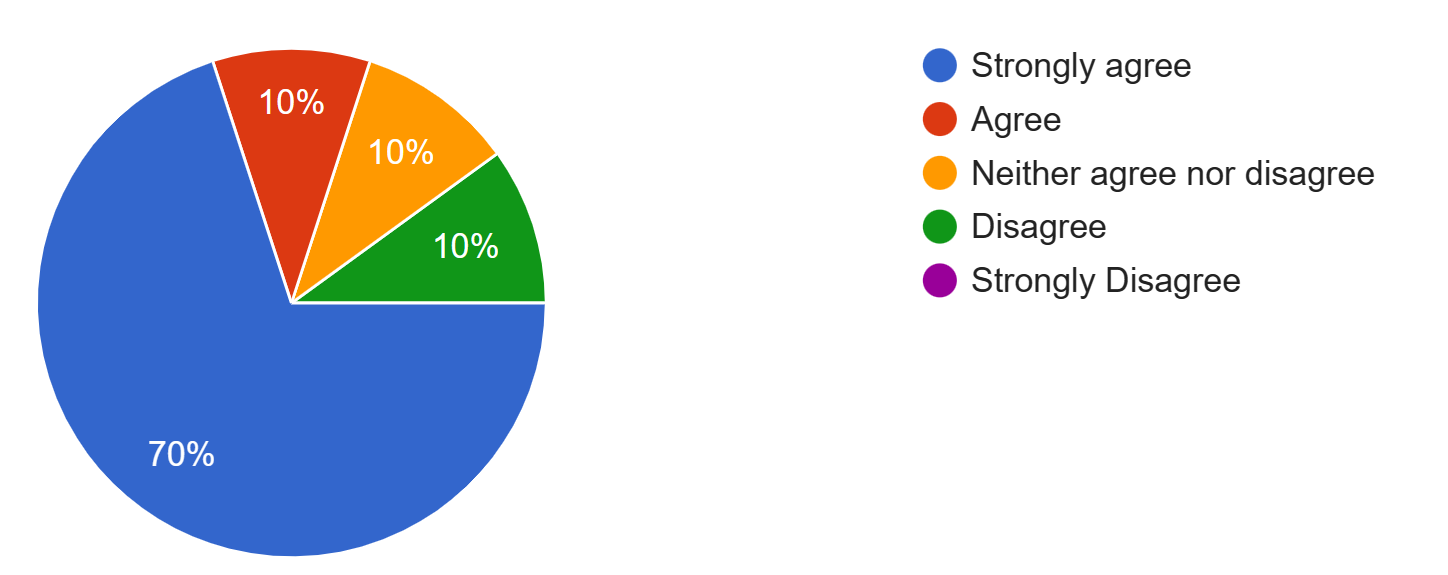


10.4 If you do not fully agree or have anything else to comment on, you can do so here.

- see comment above.
- Residues should not be divided into different categories, rather I would prefer a measurement based on a metric scale.

11.1 The DIGEST-FEES evaluates pharyngeal residue of greater than 66% ("nearly complete residue") as severe impairment of swallowing efficiency. Is this also appropriate for patients with Parkinson's disease?


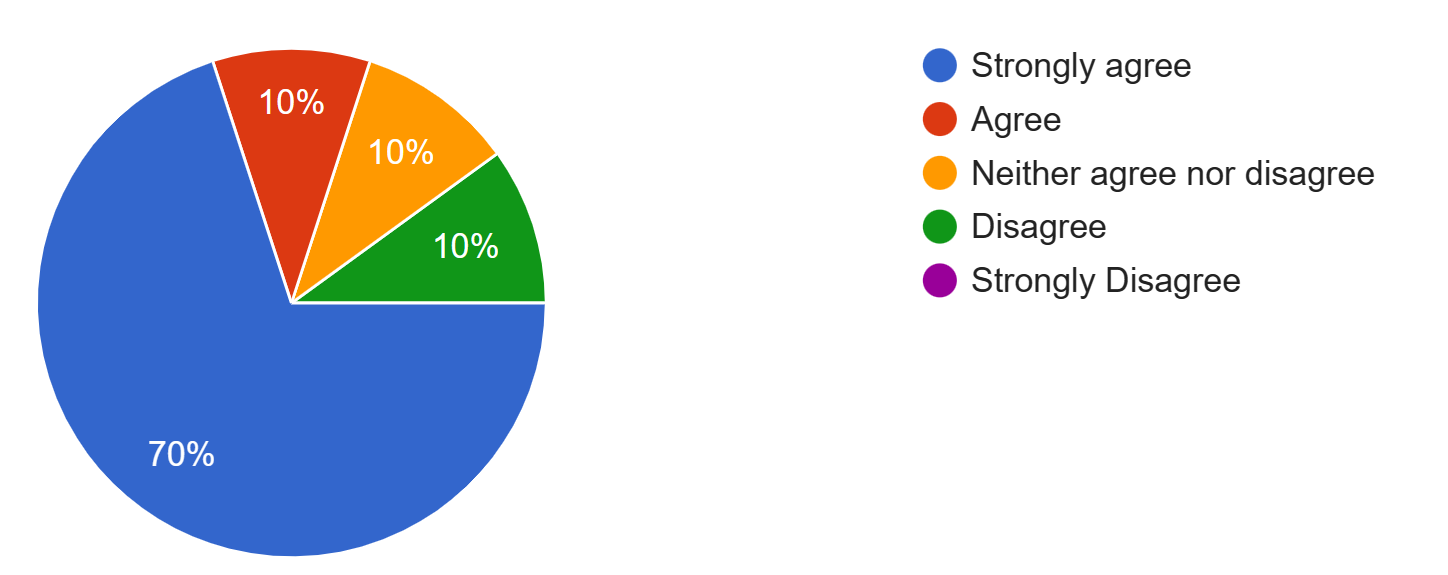


11.2 If you do not fully agree or have anything else to comment on, you can do so here.

- see comment above.
- Residues should not be divided into different categories, rather I would prefer a measurement based on a metric scale.

11.3 Is this also appropriate for patients with pharyngeal neurogenic dysphagia?


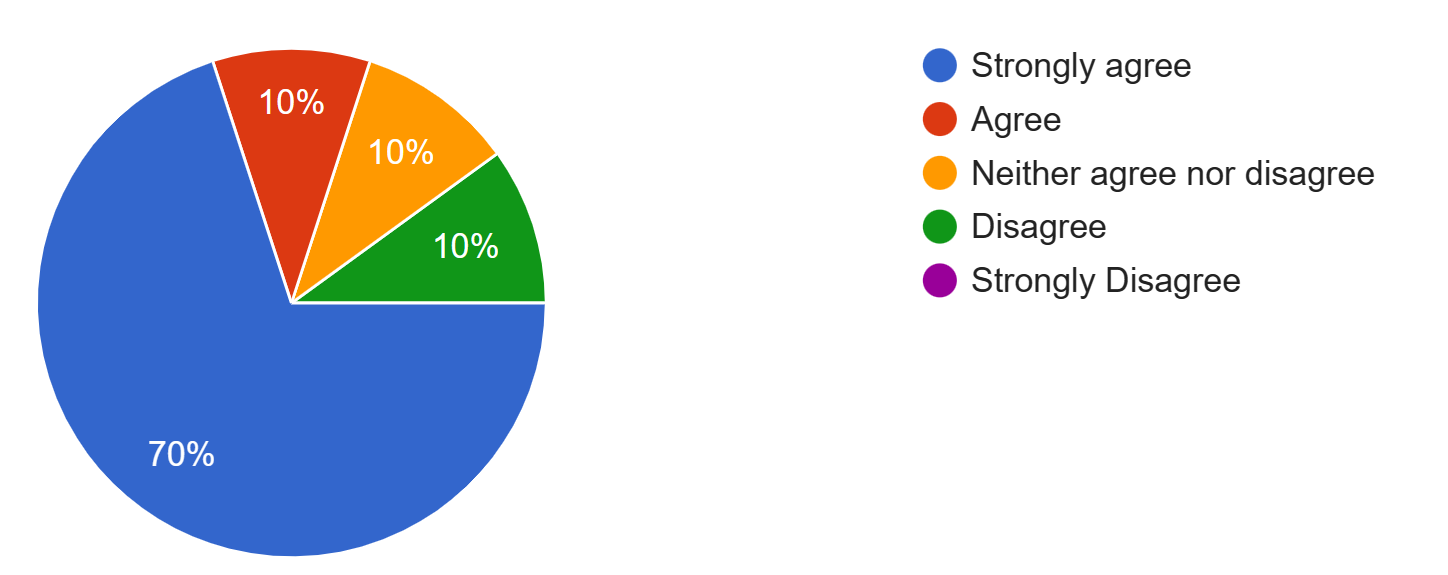


11.4 If you do not fully agree or have anything else to comment on, you can do so here.

- see comment above.
- Residues should not be divided into different categories, rather I would prefer a measurement based on a metric scale.

12.1 For moderate residue, the DIGEST-FEES assigns a higher impairment grade if the residue occurs on liquid and semisolid consistency swallowing trails, rather than only in solid consistency swallowing trails. Is this approach also appropriate for patients with Parkinson's disease?


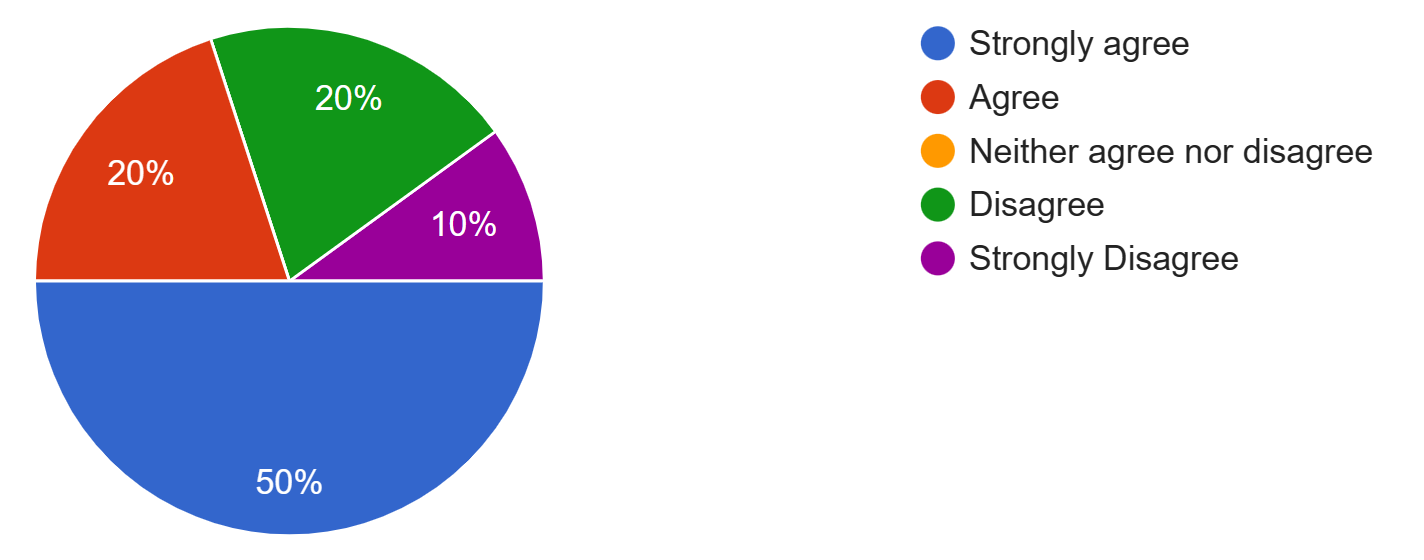


12.2 If you do not fully agree or have anything else to comment on, you can do so here.

- In patients with Parkinson's disease there should be no difference between the consistencies when classifying residue severity and overall impairment!
- This depends on what happens with the residue. Some patients deal better with certain consistencies than others. So, you can't generalise that liquid/semisolid is always worse than solid.

12.3 Is this approach also appropriate for patients with pharyngeal neurogenic dysphagia?


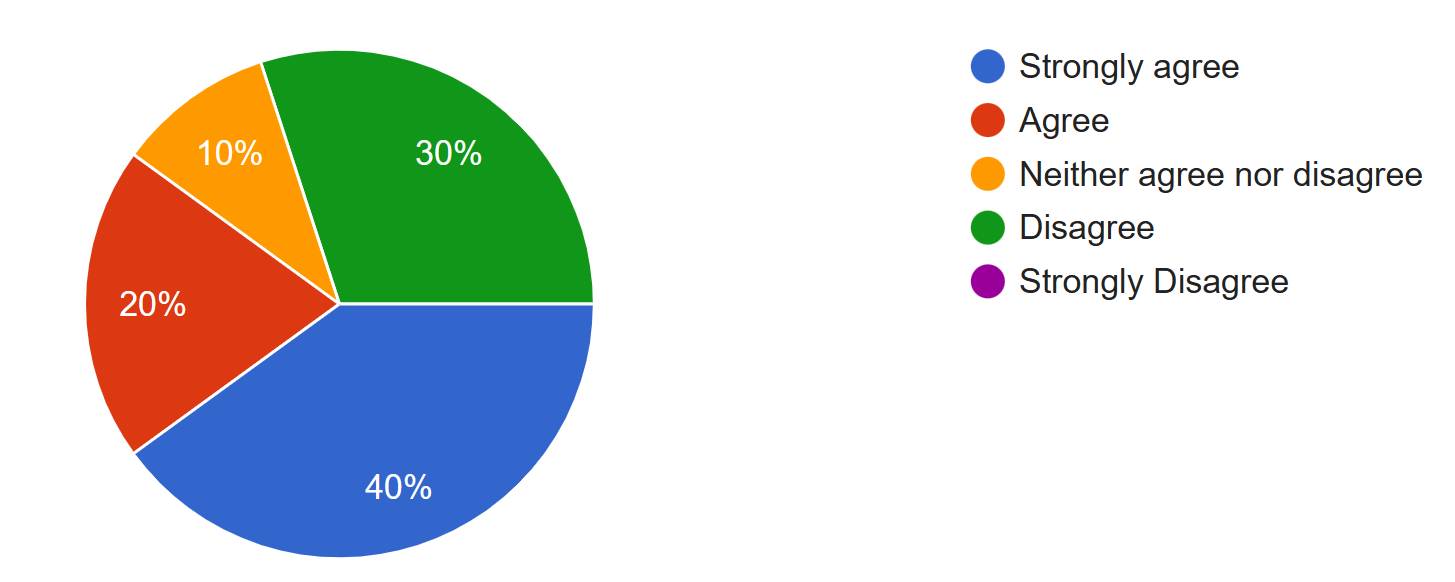


12.4 If you do not fully agree or have anything else to comment on, you can do so here.

- It depends on the different types of neurological disorders and may be different between aetiologies (for example stroke vs. dementia).
- see above.

13.1 For severe residue, the DIGEST-FEES assigns a higher level of impairment if the residue occurred on all bolus types presented, rather than just any, but not all bolus types. Is this approach also suitable for patients with Parkinson's disease?


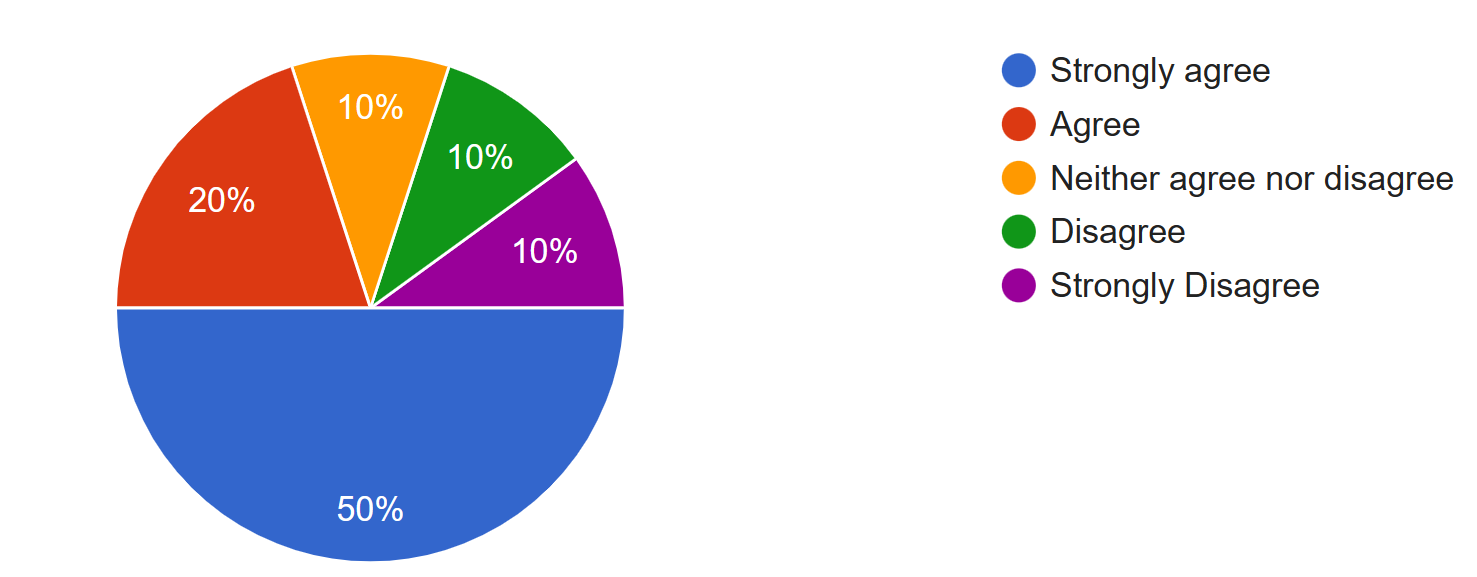


13.2 If you do not fully agree or have anything else to comment on, you can do so here.

- In patients with Parkinson's disease there should be no difference between the consistencies when classifying residue severity and overall impairment!
- see above.

13.3 Is this approach also appropriate for patients with pharyngeal neurogenic dysphagia?


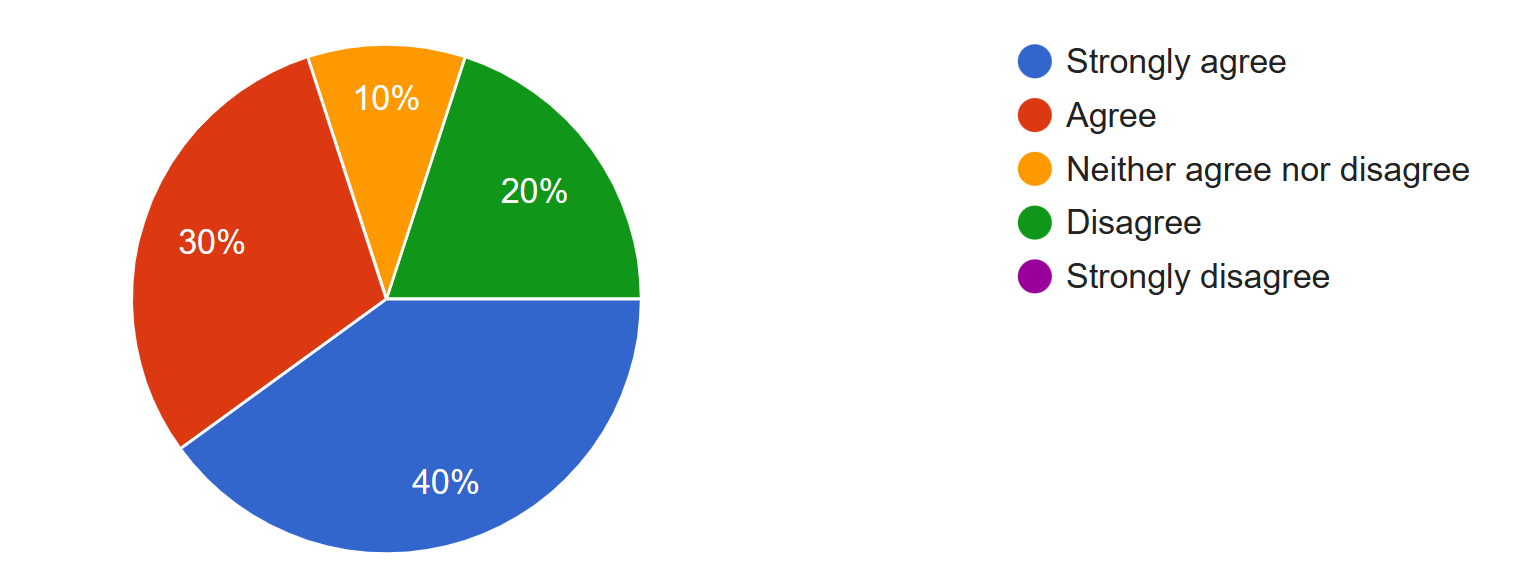


13.4 If you do not fully agree or have anything else to comment on, you can do so here.

- It depends on the different types of neurological disorders and may be different between aetiologies (for example stroke vs. dementia).
- see above.

14.1 Is the classification of impaired swallowing efficiency overall also appropriate for patients with Parkinson's disease?


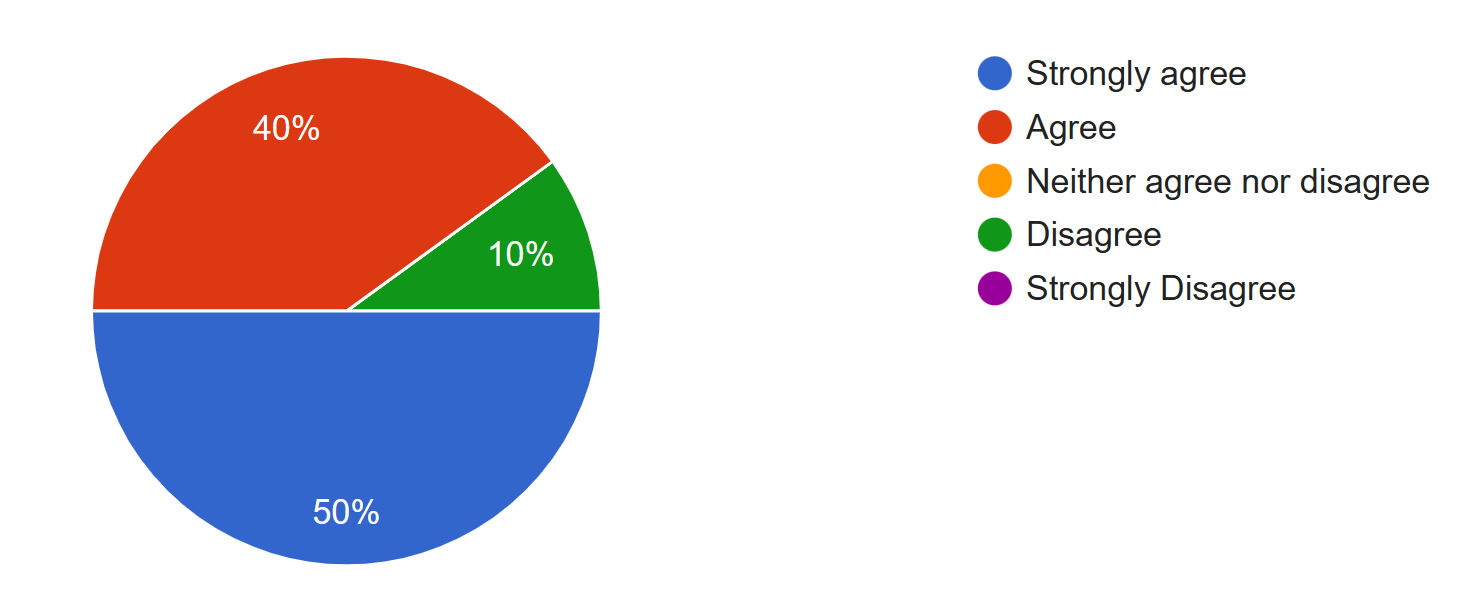


14.2 If you do not fully agree or have anything else to comment on, you can do so here.

- A fundamental problem with ordinal scaling is that the boundaries are somewhat arbitrary, and a small difference within the area of the boundary can make a big difference in the scoring. One proposed approach to this problem is VASES (Visual Analysis of Swallowing Efficiency and Safety), e.g., the scoring based on visual analogue scales. This could possibly be incorporated into the concept in the future.
- I'm not sure if it's justified to grade a single swallow with residue over 33% at solid consistency a grade 2. I think we will have to see in the future if this is empirically justified. On the other hand, residue are much less variable than penetrations and aspirations in my experience, so that the scenario of residue above 33% in a single swallowing trial should be rather rare. Therefore, I understand that frequency of events is more considered for penetrations and aspirations than for residue, where the maximum finding is more considered in the DIGEST-FEES. Overall, I think the categorization is reasonable.
- There should be no difference between consistencies!

14.3 Is the categorization of impaired swallowing efficiency overall also useful for pharyngeal neurogenic dysphagia?


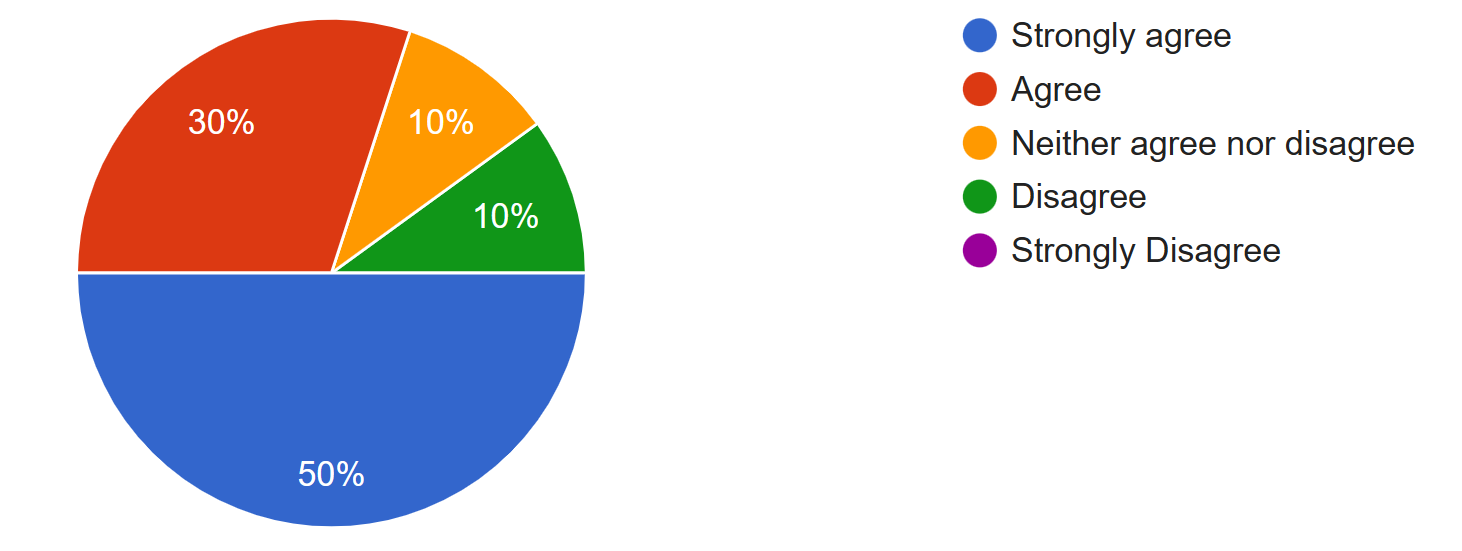


14.4 If you do not fully agree or have anything else to comment on, you can do so here.

- A fundamental problem with ordinal scaling is that the boundaries are somewhat arbitrary, and a small difference within the area of the boundary can make a big difference in the scoring. One proposed approach to this problem is VASES (Visual Analysis of Swallowing Efficiency and Safety), e.g., the scoring based on visual analogue scales. This could possibly be incorporated into the concept in the future.
- I'm not sure if it's justified to grade a single swallow with residue over 33% at solid consistency a grade 2. I think we will have to see in the future if this is empirically justified. On the other hand, residue are much less variable than penetrations and aspirations in my experience, so that the scenario of residue above 33% in a single swallowing trial should be rather rare. Therefore, I understand that frequency of events is more considered for penetrations and aspirations than for residue, where the maximum finding is more considered in the DIGEST-FEES. Overall, I think the categorization is reasonable.
- It depends on the different types of neurological disorders and may be different between aetiologies (for example stroke vs. dementia).

15.1 Is the intended interaction between swallowing safety and swallowing efficiency in determining the overall impairment level in the DIGEST-FEES appropriate in patients with Parkinson's disease?


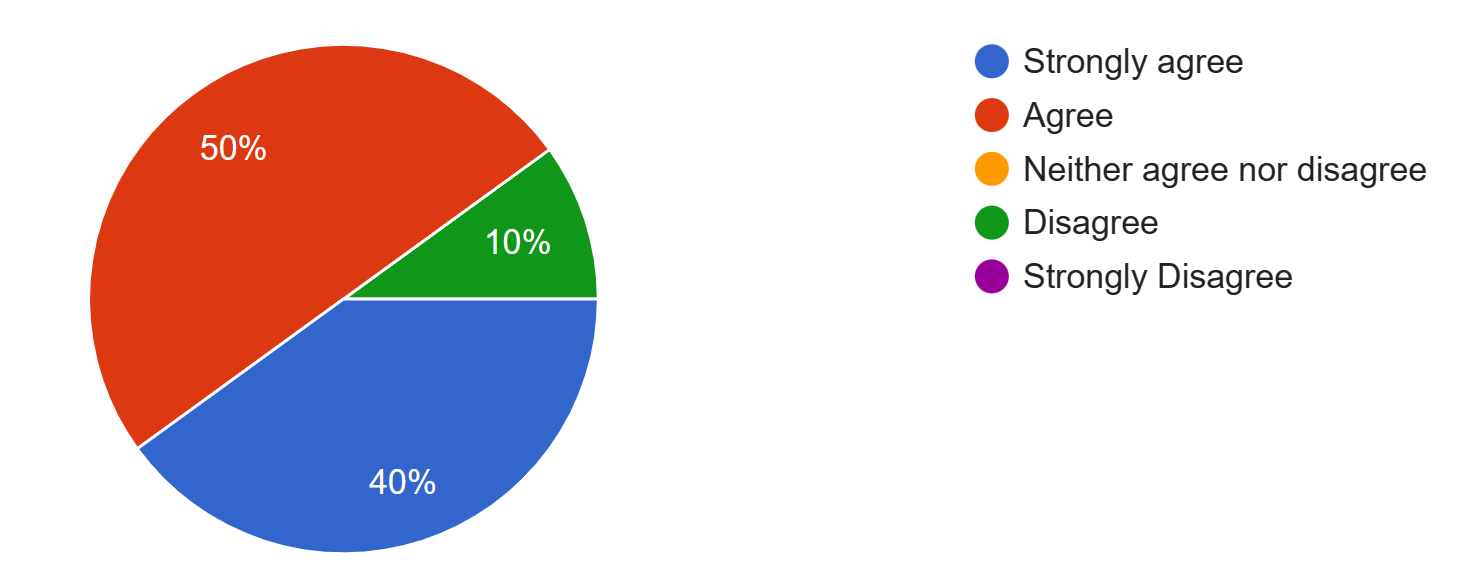


15.2 If you do not fully agree or have anything else to comment on, you can do so here.

- The relevance of penetration/aspiration is overweighted compared to residue.
- see above.

15.3 Is the intended interaction between swallowing safety and swallowing efficiency in determining the overall impairment level in the DIGEST-FEES also useful in patients with pharyngeal neurogenic dysphagia?


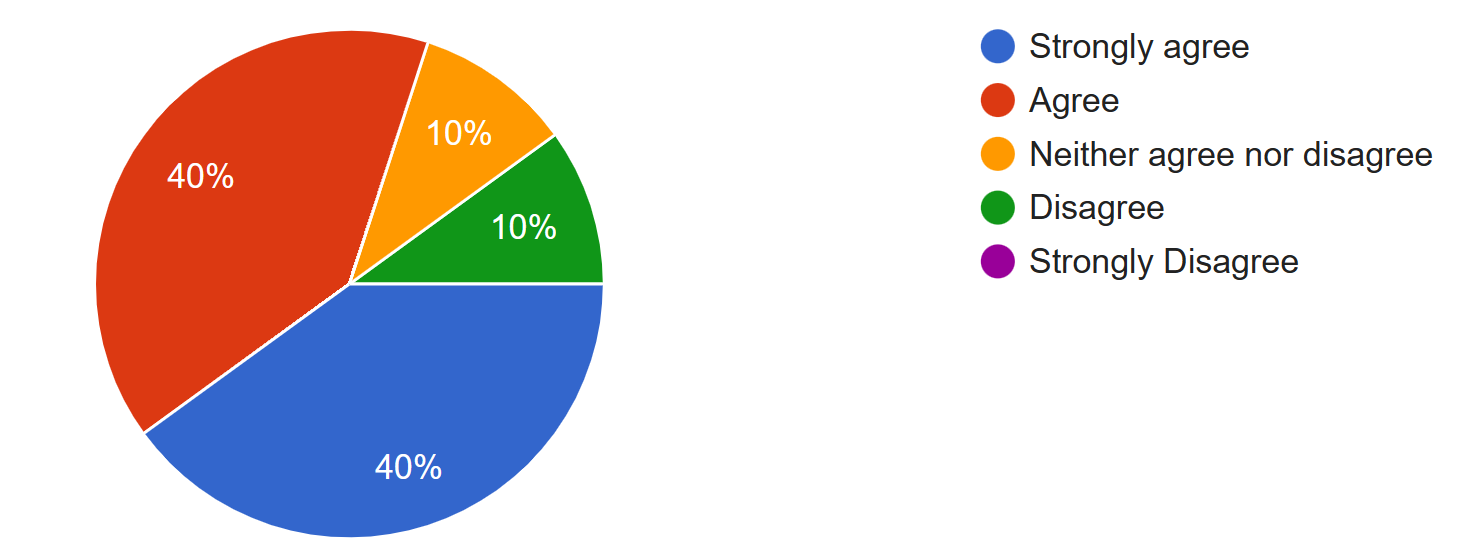


15.4 If you do not fully agree or have anything else to comment on, you can do so here.

- It depends on the different types of neurological disorders and may be different between aetiologies (for example stroke vs. dementia).
- It may not apply to all patients since neurogenic dysphagia is a heterogenic field of swallowing disturbances with quite different patterns that are not all included in this score (how could they...).
- see above.
